# The blue light-dependent LOV-protein LdaP of *Dinoroseobacter shibae* acts as antirepressor of the PpsR repressor, regulating photosynthetic gene cluster expression

**DOI:** 10.3389/fmicb.2024.1351297

**Published:** 2024-02-07

**Authors:** Saskia Pucelik, Miriam Becker, Steffi Heyber, Lars Wöhlbrand, Ralf Rabus, Dieter Jahn, Elisabeth Härtig

**Affiliations:** ^1^Institute of Microbiology, Technische Universität Braunschweig, Braunschweig, Germany; ^2^Institute for Chemistry and Biology of the Marine Environment (ICBM), Carl von Ossietzky University of Oldenburg, Oldenburg, Germany; ^3^Braunschweig Integrated Centre of Systems Biology (BRICS), Technische Universität Braunschweig, Braunschweig, Germany

**Keywords:** *Dinoroseobacter shibae*, LOV, blue light-dependent gene regulation, *Roseobacter*, transposon library screen, protein–protein interaction, aerobic anoxygenic photosynthesis, photosynthetic gene cluster

## Abstract

In the marine α-proteobacterium *Dinoroseobacter shibae* more than 40 genes of the aerobic anoxygenic photosynthesis are regulated in a light-dependent manner. A genome-wide screen of 5,605 clones from a *D. shibae* transposon library for loss of pigmentation and changes in bacteriochlorophyll absorbance identified 179 mutant clones. The gene encoding the LOV-domain containing protein Dshi_1135 was identified by its colorless phenotype. The mutant phenotype was complemented by the expression of a Dshi_1135-strep fusion protein in trans. The recombinantly produced and chromatographically purified Dshi_1135 protein was able to undergo a blue light-induced photocycle mediated by bound FMN. Transcriptome analyses revealed an essential role for Dshi_1135 in the light-dependent expression of the photosynthetic gene cluster. Interactomic studies identified the repressor protein PpsR as an interaction partner of Dshi_1135. The physical contact between PpsR and the Dshi_1135 protein was verified *in vivo* using the bacterial adenylate cyclase-based two-hybrid system. In addition, the antirepressor function of the Dshi_1135 protein was demonstrated *in vivo* testing of a *bchF-lacZ* reporter gene fusion in a heterologous *Escherichia coli*-based host system. We therefore propose to rename the Dshi_1135 protein to LdaP (light-dependent antirepressor of PpsR). Using the bacterial two-hybrid system, it was also shown that cobalamin (B_12_) is essential for the interaction of the antirepressor PpaA with PpsR. A regulatory model for the photosynthetic gene cluster in *D. shibae* was derived, including the repressor PpsR, the light-dependent antirepressor LdaP and the B_12_-dependent antirepressor PpaA.

## Introduction

Oxygenic photosynthesis mainly found in cyanobacteria, algae and plants is the major light-driven biochemical process on earth. It provides the basis for aerobic life on earth due to the conversion of carbon dioxide and water into glucose and oxygen. During this biochemical reaction light harvesting systems in combination with corresponding reaction centers, in which pigments like chlorophyll and carotene are embedded in membrane-spanning complexes, are responsible for capturing of photons and the subsequent conversion of light energy into chemical energy. However, multiple bacteria possess the evolutionary older type of aerobic anoxygenic photosynthesis (AAP; Yurkov and Beatty, 1998; Koblížek, 2015). Aside from the role of light as an energy source of the photosynthetic process, it can also act as an abiotic signal, which enables a broad variety of organisms a regulated response to changes in their environment (Montgomery, 2007; Gomelsky and Hoff, 2011; Yu and Fischer, 2019; Teixeira, 2020). The ability throughout all three kingdoms of life to sense light is enabled by so called photoreceptors. These are proteins that usually carry a light sensitive chromophore and are able to trigger different biological signal transduction cascades (Montgomery, 2007; Purcell and Crosson, 2008). Therefore, these kinds of light sensors are involved in a diverse set of biological processes that usually all need a tight regulation. During evolution, a number of different photoreceptors have evolved (Purcell and Crosson, 2008). A widely distributed type of light sensors comprises the LOV (light-oxygen-voltage) domain containing proteins that use flavin cofactors to detect light of the blue part of the spectrum (Taylor and Zhulin, 1999; Crosson et al., 2003). This type of sensors is for instance involved in phototropism, chloroplast movement in plants and circadian response in different organisms. In bacteria they were found to regulate virulence, stress response, cell attachment and photopigment biosynthesis (Briggs et al., 2001; Briggs and Spudich, 2005; Krauss et al., 2009).

The marine α-proteobacterium *Dinoroseobacter shibae*, a photoheterotrophic member of the diverse *Roseobacter* group, is able to perform aerobic anoxygenic photosynthesis (AAP; Biebl et al., 2005; Simon et al., 2017; Dlugosch et al., 2022). AAP bacteria account for up to 20% of the total bacteria in the upper layers of the ocean. Their photoheterotrophy is responsible for the respiratory organic carbon consumption of ~2.4%–5.4% of marine primary productions (Jiao et al., 2010; Ferrera et al., 2014; Koblížek, 2015; Simon et al., 2017; Giebel et al., 2019). The corresponding photosynthetic gene cluster (PGC) of *D. shibae* comprises more than 40 genes encoding enzymes of bacteriochlorophyll *a* (*bch*) and carotenoid (*crt*) biosynthesis, structural proteins of the reaction center (*pufLM, puhA*) and of light-harvesting complex I (*pufAB*), and various transcriptional regulators (*ppsR*, *tspO, ppaA*; Wagner-Dobler and Biebl, 2006; Liotenberg et al., 2008). Previous studies showed the repression of the *puf o*peron after light exposure in *Roseobacter denitrificans* and that bacteriochlorophyll synthesis was specifically blocked by blue light, indicating the role of a photoreceptor (Iba and Takamiya, 1989; Nishimura et al., 1996). An investigation of the light-dependent regulation of the PGC in *D. shibae* described the transcription of the PGC in the dark and repression of transcription after the switch to light conditions (Tomasch et al., 2011). Most of the research regarding the regulation of PGCs gene expression was performed for the two purple non-sulfur bacteria *Rhodobacter capsulatus* and *Cereibacter sphaeroides* both utilizing anaerobic anoxygenic photosynthesis. In these organisms an oxygen-dependent regulation of the PGC is mediated by the two-component systems named PrrA/B or RegA/B, and the oxygen sensor protein FnrL (Eraso and Kaplan, 1994; Bauer et al., 2003; Eraso et al., 2008; Bauer et al., 2009). In addition, there is a redox-responding aerobic repressor of photopigment, light-harvesting and cytochrome biosynthesis genes called CrtJ in *R. capsulatus* or PpsR in *C. sphaeroides* (Penfold and Pemberton, 1994; Ponnampalam et al., 1995; Elsen et al., 2005; Yin et al., 2012). In these species, PpsR/CrtJ is the main regulator of the photosynthetic gene cluster repressing transcription under aerobic conditions (Penfold and Pemberton, 1994; Ponnampalam et al., 1995). The DNA-binding activity of the CrtJ/PpsR proteins is highly regulated by oxygen tension via the formation of intramolecular disulfide bonds between conserved cysteine residues resulting in the repression of corresponding gene expression by a CrtJ/PpsR tetramer (Masuda and Bauer, 2002; Cheng et al., 2012, 2014). CrtJ/PpsR binds to the target sequence TGT-N_12_-ACA, which is located in tandem upstream of the *puc* and several *crt* and *bch* operons in *R. capsulatus* and *C. sphaeroides* (Ponnampalam and Bauer, 1997). This target sequence was also found in the promoter regions of several operons of the *D. shibae* PGC (Tomasch et al., 2011). Interestingly, in *D. shibae* no oxygen-dependent regulation of the PGC was observed (Ebert et al., 2017). Moreover, in other purple bacteria including *D. shibae* transcriptionally linked *aerR*/*ppaA* genes were always found upstream of the *ppsR* gene (Dong et al., 2002; Gomelsky et al., 2003). These PpaA/AerR protein homologs are characterized by the presence of a cobalamin (B_12_)-binding domain. In addition, B_12_ regulates photosystem gene expression in *R. capsulatus* via the CrtJ antirepressor AerR (Cheng et al., 2014). The homologous protein PpaA from *C. sphaeroides* binds cobalamin and is able to interact with PpsR as an antirepressor activating expression of the photosynthesis genes *in vivo* (Vermeulen and Bauer, 2015). For *C. sphaeroides* in addition to PpaA a second regulatory protein named AppA was described. Both, PpaA and AppA independently form a complex with PpsR in a redox-dependent manner, which abolishes the binding of PpsR to its target promoters (Masuda and Bauer, 2002; Winkler et al., 2013; Fang and Bauer, 2017). AppA of *C. sphaeroides* is a BLUF (sensor of blue light using FAD) domain containing blue light-responsive photoreceptor and its conformational change after blue light illumination is proposed to weaken the DNA binding affinity of the AppA-PpsR_2_ complex (Masuda and Bauer, 2002). Purple photosynthetic bacteria containing the PpsR/CrtJ repressor family have one or more antirepressor proteins that sense the presence of cobalamin, heme and light. In this study, we describe the functional characterization of the newly identified LOV-protein Dshi_1135 with an essential role for light-dependent regulation of the PGC in *D. shibae*.

## Results

### High-throughput screening for mutants defective in photosynthetic pigment formation

**Figure 1:**
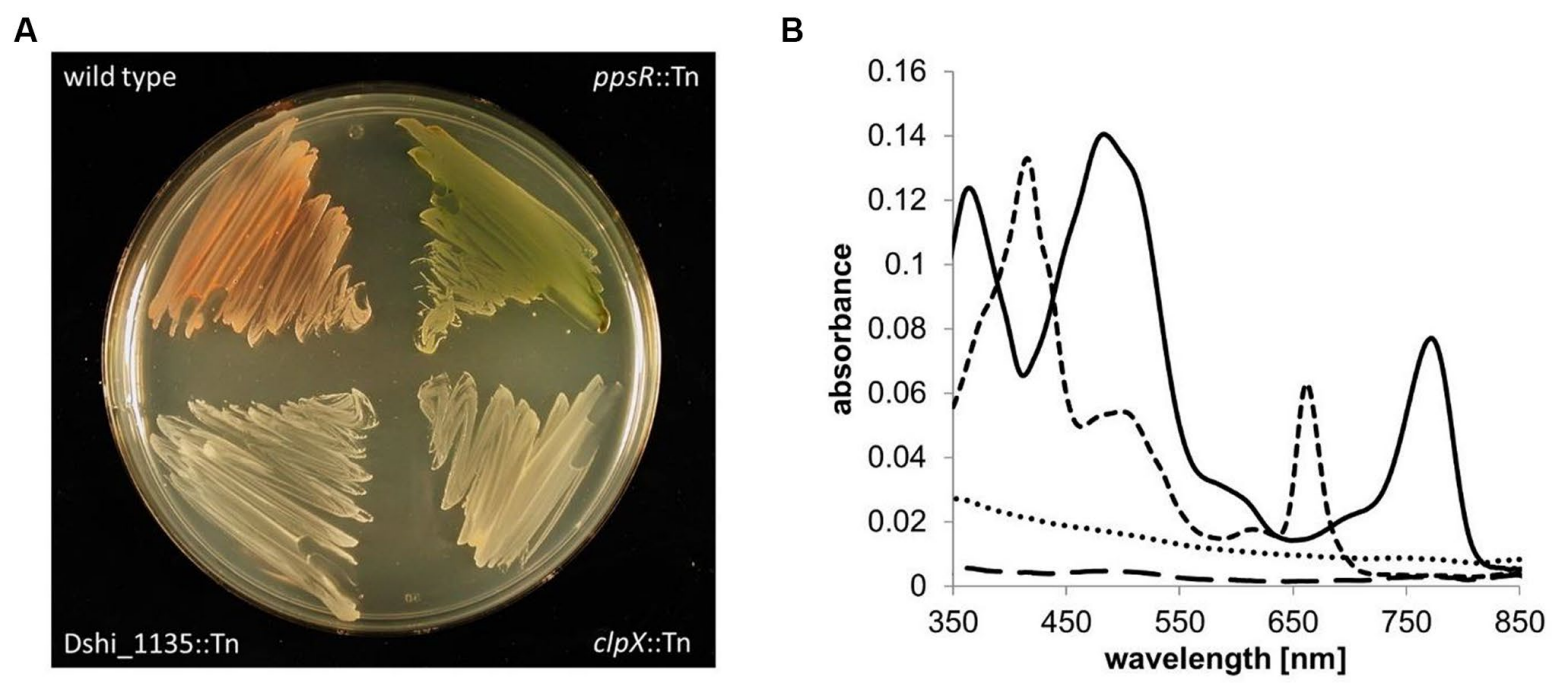
Effects of gene inactivation on photopigment abundance. **(A)** Pigmentation phenotype of the *Dinoroseobacter shibae* wild type strain and the newly identified transposon mutant strains *ppsR*::Tn, Dshi_1135::Tn and *clpX*::Tn. **(B)** Photopigments were extracted from bacteria grown in the dark and absorption was measured by UV/vis spectroscopy. Shown are the spectra of the *D. shibae* wild type strain (solid line) and the *ppsR*::Tn (dashed line), Dshi_1135::Tn (dotted line), *clpX*::Tn (loosely dashed line) mutant strains. Absorption at 374 and 780 nm obtained from the wild type strain is derived from bacteriochlorophyll, absorption between 450 to 570 nm from spheroidenone. Absorption at 418 and 680 nm observed in the *ppsR*::Tn mutant strain is probably due to the accumulation of chlorophyllide, a precursor in the bacteriochlorophyll biosynthetic pathway.

In order to identify genes for missing parts of the bacteriochlorophyll biosynthesis pathway, photosynthetic apparatus formation and corresponding regulatory compounds, we established a plate screening assay for our previously described *D. shibae* transposon library (Ebert et al., 2013). First, mutants were identified by an altered pigmentation phenotype. Furthermore, *in vivo* UV/vis spectra of transposon mutants grown in 96 well plates were recorded and analyzed for changes in pigment-dependent absorption. The *D. shibae* DFL12^T^ wild type strain showing the typical pink pigmentation with an absorption peak between 450 and 570 nm derived from spheroidenone and the typical absorption peaks for bacteriochlorophyll *a* at 374 nm (Soret peak), 590 nm (Q_x_), 804 nm and 868 nm (Q_Y_) from the light harvesting complex II served as control (Supplementary Figure S1). The *D. shibae* mutant strain *acsF*::Tn with a transposon integrated into the gene encoding the magnesium-protoporphyrin IX monomethyl ester cyclase, served as standard for the accumulation of the fluorescent magnesium-protoporphyrin IX monomethyl ester (MPE). Accumulation of MPE, indicative for the accumulation of bacteriochlorophyll biosynthesis intermediates, was monitored by autofluorescence of the corresponding colonies under blue light illumination which resulted in a distinct absorption peak at 416 nm while at the same time bacteriochlorophyll absorption was gone (Supplementary Figure S1). In total, 5,605 clones of the transposon library, representing 2,735 different gene loci corresponding to 65% of the whole genome, were screened for changes in pigmentation. Transposon integration sites were determined using arbitrary PCR as described before (Ebert et al., 2013). Overall, 34 transposon mutants were found accumulating MPE and 145 transposon mutants showed reduced or abolished Bchl *a* absorption peaks at 804 and 868 nm. As a result of the transposon library screening, 10 clones with inactivated genes of Bchl *a* biosynthesis were identified, confirming the suitability of the experimental approach: Dshi_2636 (*bchJ*, encoding the bacteriochlorophyll 4-vinyl reductase), Dshi_2637 (*bchE*, encoding the magnesium-protoporphyrin IX monomethyl ester anaerobic oxidative cyclase), Dshi_3517 (*bchX*, encoding the chlorophyllide reductase iron protein subunit X), Dshi_3518 (*bchY*, encoding the chlorophyllide reductase subunit Y), Dshi_3519 (*bchZ*, encoding the chlorophyllide reductase subunit Z), Dshi_3528 (*bchP*, encoding the geranylgeranyl bacteriochlorophyll reductase), Dshi_3533 (*bchF*, encoding the bacteriochlorophyllide hydratase), Dshi_ 3,536 (*bchH*, encoding the magnesium chelatase subunit H), Dshi_3535 (*bchB*, encoding the light-independent protochlorophyllide reductase subunit B) and Dshi_3544 (*acsF*, encoding the magnesium-protoporphyrin IX monomethyl ester cyclase). Two inactivated genes encoding proteins of the photosynthetic reaction center Dshi_3540 (*puhA*::Tn) and Dshi_3541 (*puhB*::Tn) were identified by changes in pigmentation and accumulation of MPE (Table 1). Moreover, two transposon mutants of genes encoding potential regulators of Bchl *a* biosynthesis genes Dshi_3531 (*ppsR*::Tn) and Dshi_3532 (*ppaA*::Tn) were identified. The *ppsR*::Tn mutant strain showed a greenish color. Most likely, the observed peaks at 418 and 660 nm resulted from chlorophyllide *a* absorbance, a late intermediate in Bchl *a* biosynthesis and the direct precursor of Bchl *a*. Similar peaks were obtained in a *R. capsulatus* mutant strain when the genes *bchF* and *bchZ* were disrupted (Bollivar et al., 1994; Figure 1). These results clearly showed the power of the screening approach by identifying important known compounds of the bacteriochlorophyll biosynthesis and corresponding regulatory components.

**Table 1:** Transposon mutant strains identified by altered pigmentation, MPE accumulation and changes in bacteriochlorophyll *a* absorption measured by *in vivo* UV/vis spectroscopy.

Gene loci inactivated by transposon insertion	Pigmentation		Visible spectra
	on MB	in ASM	
Wild type	Pink	Pink	Bchl^1^
**Genes of bacteriochlorophyll biosynthesis**			
Dshi_2636 (*bchJ*::Tn)	Pink	Pink	Bchl^1^
Dshi_2637 (*bchE*::Tn)	Pink	Pink	Bchl^1^ + MPE^2^
Dshi_3517 (*bchX*::Tn)	Yellow	Beige	-
Dshi_3518 (*bchY*::Tn)	Yellow	Beige	-
Dshi_3519 (*bchZ*::Tn)	Yellow	Beige	-
Dshi_3528 (*bchP*::Tn)	Pink	Pink	Bchl^1^
Dshi_3533 (*bchF*::Tn)	Yellow	Beige	-
Dshi_3535 (*bchB*::Tn)	Yellow	Beige	MPE^2^
Dshi_3536 (*bchH*::Tn)	Beige	Beige	-
Dshi_3544 (*acsF*::Tn)	Yellow	Yellow	MPE^2^
**Genes of the photosynthetic apparatus**			
Dshi_3540 (*puhA*::Tn)	Orange	Beige	MPE^2^
Dshi_3541 (*puhB*::Tn)	Yellow	Yellow	Bchl^1^ + MPE^2^
**Genes encoding regulators of the PGC**			
Dshi_1135(*ldaP*::Tn)	Pink	White	-
Dshi_3531 (*ppsR*::Tn)	Green	Green	MPE^2^ + 665 nm
Dshi_3532 (*ppaA*::Tn)	Pink	Pink	Bchl^1^ + MPE^2^
**Proteolyses genes**			
Dshi_1387 (*clpX*::Tn)	Yellow	Beige	MPE^2^
Dshi_1388 (*clpP*::Tn)	Pink	White	Bchl^1^

^1^Absorbance at 374 nm (Soret), 590 nm (QX) und 804 bzw. 868 nm (QY), typical for Bchl a; ^2^Absorbance at 416 nm typical for MPE.

In addition, gene loci Dshi_1387, Dshi_1388 and Dshi_1135 were identified by the screen. The Dshi_1187::Tn and Dshi_1135::Tn mutant strains showed neither pigmentation on agar plates nor absorption during UV/vis spectroscopy (Figure 1). The gene Dshi_1387 encodes the ATP-binding subunit ClpX of the ATP-dependent ClpXP protease, which will be investigated in a different study. The Dshi_1135 gene codes for a 338 amino acid protein with a putative N-terminal PAS photoreceptor and a C-terminal histidine kinase domain (NCBI reference: WP_012177807.1). The photoreceptor domain showed a high degree of amino acid sequence identity to LOV (light-oxygen-voltage) domain proteins. First, we checked the dependence of the phenotype on the single integration in the Dshi_1135 gene by complementation of the Dshi_1135::Tn mutant strain by a Dshi_1135 gene copy *in trans.* For this purpose, the Dshi_1135 mutant strain was transformed with the plasmid pRhoKS_Dshi_1135-Strep encoding the Dshi_1135 gene fused to DNA for a Strep-Tag under the control of the constitutive *aphII* promoter. Complementation resulted in the restoration of the wild type pigmentation (Supplementary Figure S2).

### Dshi_1135, PpaA, and PpsR are regulators for transcriptional activation of the photosynthetic gene cluster in *Dinoroseobacter shibae*

**Figure 2:**
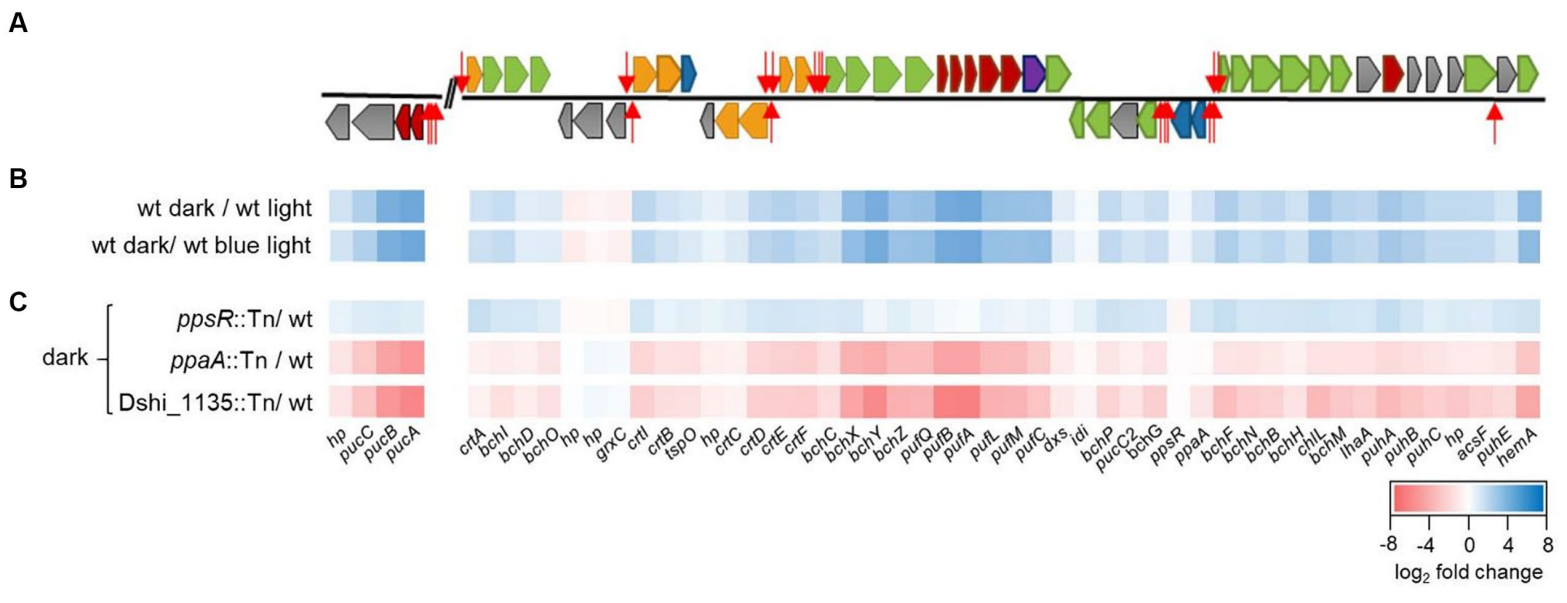
Transcriptome data of the photosynthetic gene cluster (PGC) for the wild type strain *Dinoroseobacter shibae* DFL12^T^ and the Dshi_1135::Tn, the *ppsR*::Tn and the *ppaA*::Tn mutant strains, respectively. **(A)** Schematic representation of the PGC (Dshi_3501–Dshi_3547) and the *puc* operon (Dshi_2897–Dshi_2900). Red indicates structural components of the photosystem; green, Bchl *a* biosynthesis genes; orange, spheroidenone biosynthesis genes; blue, regulatory proteins; velvet, cytochrome; gray, assembly proteins or hypothetical proteins. Potential PpsR binding sites are indicated by red arrows (modified after Tomasch et al., 2011). **(B)** Transcriptome analyses comparing transcript levels of the *D. shibae* wild type strain derived from cells cultivated in the dark and under high light or blue light conditions. Data are submitted to GEO database (GSE244289). **(C)** Transcriptome analyses comparing transcript levels of *D. shibae* wild type strain with mutant strains *ppsR*::Tn, *ppaA*::Tn or Dshi_1135::Tn, respectively, all cultivated in the dark. Heat maps showing gene expression of the photosynthetic gene cluster. The color bar represents the expression level in log_2_ fold scale. Red indicates relatively low expression levels in the mutant strains in comparison to the wild type strain; blue indicates relatively high expression levels. Gene names are indicated below the respective ORFs. Genes encoding hypothetical proteins are indicated by hp.

The PGC of *D. shibae* comprises about 40 different genes encoding enzymes and proteins involved in Bchl (*bch*) and carotenoid (*crt*) biosynthesis, assembly of the reaction center and light harvesting complex (*puf, puh*) and regulators of the photosynthesis machinery (Zheng et al., 2011; Nagashima and Nagashima, 2013; Liu et al., 2019; Figure 2A). Expression of the PGC together with the *puc* operon in the *D. shibae* wild type strain was previously found upregulated under dark growth conditions and reduced under light conditions (Tomasch et al., 2011; Figure 2B). Expression analysis under blue light conditions showed a similar pattern to light conditions and was found to be lower compared to the dark growth conditions (Figure 2B). We therefore concluded a blue light-dependent regulatory effect. Next, the transcript levels of the Dshi_1135:Tn mutant strain were compared with the wild type strain after growth in the dark. In total 437 genes were found to be higher and 122 genes lower expressed in the Dshi_1135::Tn mutant (cut off at log 2-fold change ≥ 0.8). Among the genes with decreased expression all photosynthetic genes localized in the PGC and the *puc* operon were found. Maximal changes up to log_2_FC of −6.75 were reached (Figure 2C). These results agree well with the pale pigmentation phenotype and the reduced bacteriochlorophyll content of the Dshi_1135::Tn mutant strain. Furthermore, they clearly indicate a regulatory function of the Dshi_1135 LOV protein for the activation of the PGC in *D. shibae.* In addition, mutant strains of the regulatory proteins PpsR (*ppsR*::Tn) and PpaA (*ppaA*::Tn) were analyzed. Inactivation of the *ppsR* gene resulted in an increased expression of all genes of the PGC and the *puc* operon under dark growth conditions compared to the wild type strain. The gene deduced amino acids sequence of *D. shibae* PpsR shares about 44% identity with the amino acid sequences of CrtJ of *R. capsulatus* and 49% with those of PpsR of *C. sphaeroides* (Supplementary Figure S3). Using the VirtualFootprint tool of the PRODORIC database^1^ and the DNA binding sequences of the PpsR repressor from *C. sphaeroides*, several PpsR binding sites were identified upstream of the PGC operons in *D. shibae* (Figure 2A; Tomasch et al., 2011; Dudek and Jahn, 2022). Since inactivation of the *ppsR* gene in *D. shibae* resulted in increased gene expression of the PGC and potential binding sites for PpsR were identified within the promoter sequences, a repressor function of *D. shibae* PpsR for the PGC was concluded. The *ppaA* and *ppsR* genes are forming an operon regulated by the *ppaA* promoter. Comparing transcript levels of the *ppaA*::Tn mutant strain with the wild type strain showed a decreased expression of the PGC in the *ppaA*::Tn mutant strain in the dark (Figure 2C). This result assigned an antirepressor function to *D. shibae* PpaA comparable to the homologous proteins PpaA of *C. sphaeroides* and AerR of *R. capsulatus* (Dong et al., 2002; Gomelsky et al., 2003). In conclusion, screening of the *D. shibae* transposon mutant library identified the Dshi_1135 gene product as a new light-dependent regulator of the PGC in *D. shibae* in the framework of the known PpsR/PpaA repressor/antirepressor system.

### Dshi_1135 is an FMN-dependent blue light-responsive photosensor

**Figure 3:**
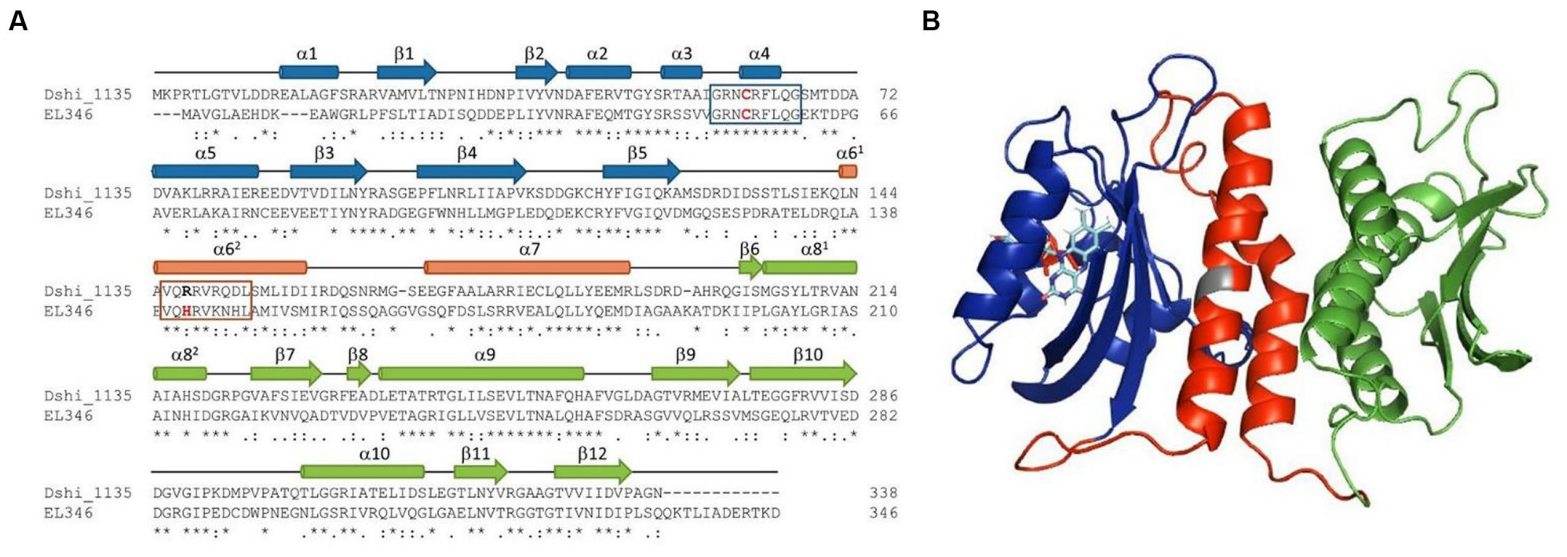
Comparison of the Dshi_1135 and the EL346 LOV-histidine kinase. **(A)** Amino acid sequence alignment of the deducted Dshi_1135 sequence with the LOV histidine kinase EL346 of *Erythrobacter litoralis.* Structural elements are derived from the EL346 structure PDB 4R3A. The LOV, DHp and CA domains are highlighted in blue, red and green, respectively. The highly conserved GRNCRFLQ sequenced is boxed in blue and the photoactive cysteine is labeled in red. The H box containing the phosphor-accepting histidine residue for autophosphorylation activity of EL346 is boxed in red. The arginine residue which replaced the histidine in the Dshi_1135 sequence is marked by a bold letter. **(B)** Structural model of Dshi_1135 obtained by homology modelling according to EL346 structure 4R3A (PDB) using SWISS-MODEL and PyMOL (Arnold et al., 2006; Schrödinger, 2015; Waterhouse et al., 2018). The LOV, DHp and CA domains are highlighted in blue, red and green, respectively. The arginine residue within alpha helix 6 at amino acid position 148 of Dshi_1135 is shown gray.

**Figure 4:**
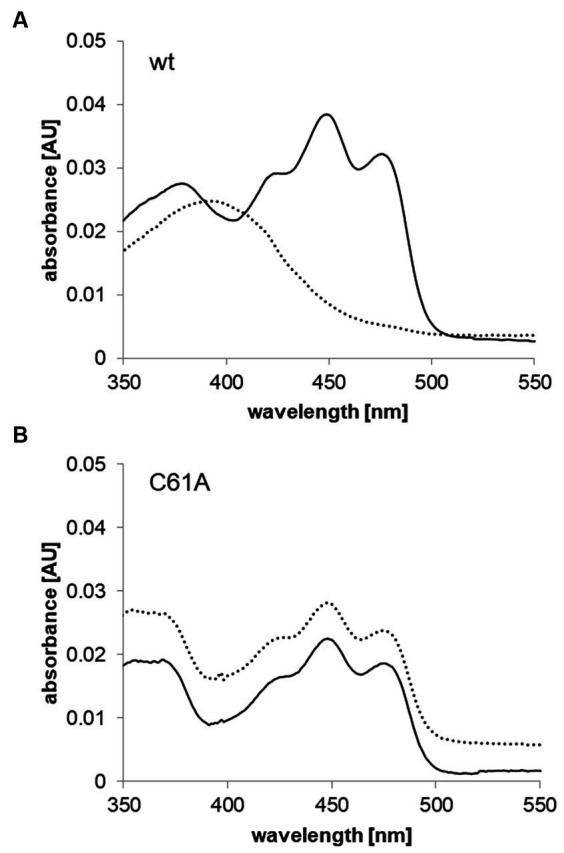
UV/vis spectra of purified and FMN reconstituted wild type Dshi_1135 and mutant variant Dshi_1135C61A under dark and blue light conditions. **(A)** Ground state absorbance spectra of purified and FMN reconstituted TRX-StrepII-Dshi_1135 protein were recorded under dim red light (black line) and showed an absorption maximum at 450 nm and the vibronic structure at 425 and 475 nm, indicative for the non-covalent binding of the FMN cofactor in the dark state. The same sample was illuminated with blue light (λ = 450 nm) for 5 min and measured again in the UV/vis spectrophotometer (dashed line). An absorbance maximum at 390 nm indicated the conversion of Dshi_1135 into the signaling state. **(B)** Spectra of purified and FMN reconstituted TRX-StrepII-Dshi_1135C61A mutant protein were recorded under dim red light (black line) and after illumination with blue light (λ = 450 nm) for 5 min (dashed line). No difference, consequently no signaling state was observed.

In order to clarify the functional role of Dshi_1135 in light-dependent regulation of the PGC expression a biochemical characterization of the protein was initiated. During an initial BLAST analysis using the Dshi_1135 amino acid sequence as query, the protein EL346 (locus tag ELI_04860) of the related organism *Erythrobacter litoralis HTCC2594* was identified with an amino acid identity of 42%. The EL346 protein is a well-studied monomeric LOV HK that consists of a N-terminal LOV domain, a dimerization/histidine phosphotransfer like domain (DHpL) and a C-terminal catalytic domain (CA). EL346 is involved in general stress response in *E. litoralis* possesses a flavin mononucleotide (FMN) cofactor that undergoes a photocycle under blue light illumination, which triggers HK activity (Swartz et al., 2007; Correa et al., 2013; Rivera-Cancel et al., 2014; Losi and Gärtner, 2017). An amino acid alignment of Dshi_1135 and EL346 together with structural modeling using alphafold 2 and comparison to the structure of EL346 (PDB 4R3A) identified highly conserved structural domains and conserved sequences on the amino acid level (Figure 3). The GRNCRFLQ amino acid sequence motif was found conserved within the LOV domain of EL346 and Dshi_1135 (amino acid position 58 to 65) and harbors the essential cysteine residue for photo-adduct formation. Surprisingly, the histidine residue for autophosphorylation was not conserved comparing the DHpL domain of EL346 with Dshi_1135. Instead, Dshi_1135 carries an arginine residue at the proposed amino acid position 148 instead of the histidine in the *E. litoralis* histidine kinase EL346. In EL346 the corresponding histidine at position 142 was experimentally verified as the phosphate acceptor (Rivera-Cancel et al., 2014). In order to validate the functional role of Dshi_1135 as a blue light-sensing LOV protein, Dshi_1135 was heterologously produced in *E. coli* as a N-terminal StrepII-tagged fusion protein and purified to apparent homogeneity via affinity chromatography (Supplementary Figure S4). To identify the potential FMN cofactor, absorption spectra were recorded. The UV/vis spectrum of the purified Strep-Dshi_1135 protein showed the major peak at 450 nm and vibronic bands at around 425 nm and 475 nm, which can be attributed to a tight, non-covalent bound FMN chromophore into the non-polar binding pocket of the LOV domain (Figure 4A). Illumination of the Dshi_1135 protein with blue light led to a bleaching of the absorption peak at 450 nm, indicating the conversion of the Dshi_1135 LOV domain from the dark into the signaling state, which in turn led to the formation of a covalent cysteinyl-flavin C4(a) adduct with a new absorption peak at 390 nm (Figure 4A). This covalent bond is of transient nature since transferring the protein back into the dark reverted it into the ground state again. This observation indicated the cleavage of the cysteinyl-flavin C4(a) adduct. These measurements demonstrated that the Dshi_1135 protein is capable of a reversible blue light induced photocycle, which is characteristic for LOV proteins.

### Cysteine 61 is essential for the blue light-induced activation of Dshi_1135

To prove that the homologous cysteine at amino acid position 61 of Dshi_1135 is involved in the photo-adduct formation, the cysteine C61 of Dshi_1135 was replaced by a non-polar, non-reactive alanine residue by site-directed mutagenesis of the corresponding gene sequence. The resulting mutant protein Dshi_1135C61A was produced as a StrepII-tagged fusion protein in *E. coli*, purified and subsequently analyzed by UV/vis spectroscopy. The Dshi_1135C61A mutant showed the same ground state absorption properties in the dark with peak maxima at 450 nm as the wild type protein (Figure 4B). Accordingly, Dshi_1135C61A was still able to bind FMN non-covalently. However, after blue light irradiation of Dshi_113C61A, no spectral bleaching was observed. The mutant protein still showed an absorption maximum at 450 nm indicating that Dshi_1135C61A is no longer able to form an FMN-cysteinyl adduct. Previous studies already showed that the formation of the adduct state is blocked in cysteine-deletion mutants (Diensthuber et al., 2014). These data clearly showed that Dshi_1135 is indeed a novel blue light photosensor with the typical properties of a LOV protein and that the cysteine at position 61 in the amino acid sequence is the reactive residue for photo-adduct formation and photocycle activity.

### Regulation by Dshi_1135 is independent of autophosphorylation properties

The potential histidine kinase domain activity of Dshi_1135 to incorporate [γ-^32^P] at a specific amino acid residue was investigated by performing autophosphorylation assays under different light conditions. First, optimal concentrations and ratios of Dshi_1135 protein, [γ-^32^P] ATP and unlabeled ATP were determined. Additionally, samples were taken at various time points after reaction start. In these various experimental approaches, we failed to observe any autophosphorylation for the Dshi_1135 protein. As an alternative assay Phos-tag™ SDS-PAGE was performed to detect autophosphorylation (Barbieri and Stock, 2008). Purified and reconstituted TRX-StrepII-Dshi_1135 protein was mixed with different ATP concentrations (1 μM, 10 μM, 100 μM and 1 mM) and either incubated in the dark or under constant blue light irradiation (λ = 467 nm) overnight. Samples were subsequently analyzed on 10% SDS gels supplemented with 100 μM Phos-tag™ and MnCl_2_ ions. While the control protein α-casein showed a shift in the phosphorylated form, only one main band was detected for the Dshi_1135 protein at the level of the unphosphorylated protein (Supplementary Figure S4). Together with the finding, that Dshi_1135 harbors an arginine residue instead the proposed phosphate-accepting histidine residue at amino acid position 148, we concluded that Dshi_1135 is a blue light-dependent LOV protein without histidine kinase function.

### Transcriptional regulation of the photosynthetic gene cluster by Dshi_1135, PpaA, and PpsR

**Figure 5:**
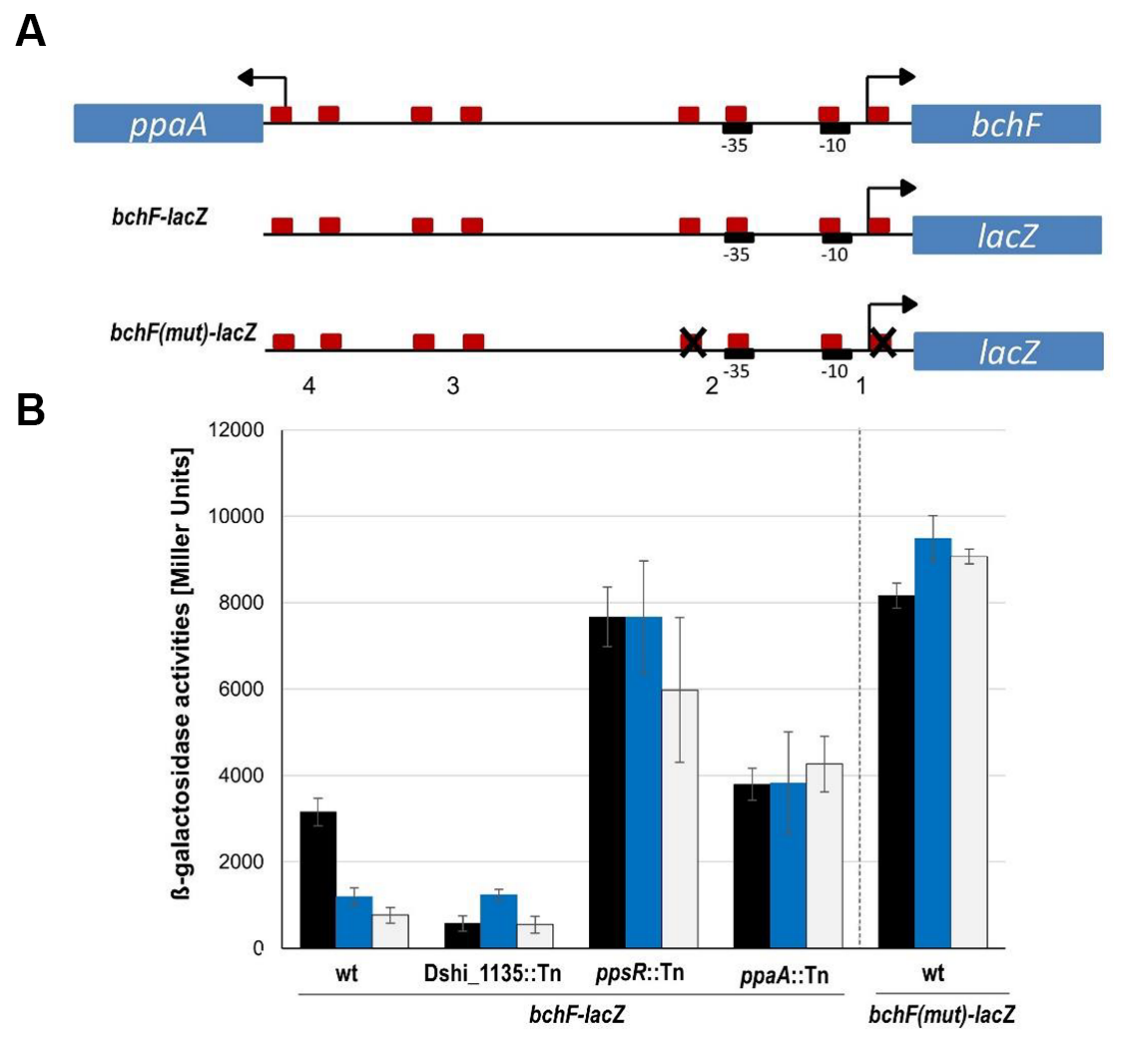
Light-dependent expression analyses of the *bchF*-*lacZ* and *bchF(mut)-lacZ* reporter gene fusions in the *Dinoroseobacter shibae* wild type strain and the Dshi_1135::Tn, *ppsR*::Tn and *ppaA*::Tn mutant strains. **(A)** Schematic representation of the genomic organization of the *bchF* and *ppaA* promoter region and the *bchF*-*lacZ* and *bchF(mut)-lacZ* reporter gene fusions tested. Red boxes indicate PpsR binding half sites, −10 and − 35 promoter regions are depicted as black boxes and the potential transcriptional start sites are indicated by arrows. **(B)** Plasmids pBBR1LIC_*bchF*-*lacZ* or pBBR1LIC*_bchF(mut)lacZ* were transformed into the *D. shibae* wild type strain and Dshi_1135::Tn, *ppsR*::Tn and *ppaA*::Tn transposon mutant strains by biparental mating, using *E. coli* ST18 as donor strain. The newly generated corresponding *D. shibae* strains DS181, DS187, DS188, DS189 and DS190 were grown under dark, blue light (467 nm) and white light conditions in biological triplicates. Samples of 0.5 mL bacterial cultures were taken in the mid-log growth phase in technical triplicates after incubation for 16–18 h at 25°C and β-galactosidase activities were determined. Mean values of the β-galactosidase activities and the corresponding standard deviations are indicated in Miller Units.

The *bchF* and the *ppaA* genes are divergently transcribed and located within the photosynthetic gene cluster (PGC). Thus, the promoter regions are overlapping and within this intergenic region four potential PpsR binding sites were identified by Virtual Footprint analyses (Figure 5A). In order to study light-dependent gene regulation in detail and the role of the transcriptional regulators PpsR, PpaA and Dshi_1135, a *bchF*-*lacZ* reporter gene fusion was constructed. The *bchF* gene is the first gene of the *bchFNBHLM* operon encoding proteins of the bchl *a* biosynthesis. A 329 bp fragment of the *bchF* promoter region, corresponding to DNA sequences from −289 to +41 with respect to the translation start of the *bchF* gene was fused to the *E. coli lacZ* reporter gene and introduced into the *D. shibae* wild type, the Dshi_1135::Tn, the *ppsR*::Tn and the *ppaA*::Tn *D. shibae* mutant strains. Strains were grown under dark, blue light and white light conditions and β-galactosidase activities were determined in the mid log phase. Under dark conditions approximately 3,000 Miller Units were recorded for the wild type strain and a 3 to 4-fold lower expression was measured under blue light (approx. 1,100 Miller Units) and white light conditions (about 750 Miller Units), respectively (Figure 5B). Obtained results nicely reflected the results obtained by the transcriptome analyses. For the *D. shibae* Dshi_1135::Tn mutant strain grown in the dark, the expression level of the *bchF*-*lacZ* reporter gene fusion resulted only in 575 Miller units. Compared to the wild type level this reflected a 6-fold lower expression in the dark, indicating a crucial role of Dshi_1135 for *bchF*-*lacZ* activation or derepression upon dark conditions. In contrast, β-galactosidase activities under blue light and white light conditions with approx. 1,200 and 550 Miller units, respectively, were comparable to the data measured for the wild type strain.

The *D. shibae ppsR*::Tn mutant strain showed a very strong expression of the *bchF*-*lacZ* fusion under dark and blue light conditions with up to 7,700 Miller units (Figure 5B). Expression under white light was only slightly weaker with 6,000 Miller Units. The expression of *bchF*-*lacZ* in the *D. shibae ppsR*::Tn mutant strain was about 8-fold stronger in white light and about 6-fold stronger under blue light conditions compared to the wild type strain (Figure 5B). Even under dark conditions a 2.5-fold higher expression of the *bchF-lacZ* was determined in the *ppsR*::Tn mutant strain (Figure 5B). These results clearly confirmed a repressor function of the PpsR regulator in *D. shibae*.

For the *ppaA*::Tn transposon mutant strain an expression level of the *bchF-lacZ* construct of about 3,800 Miller Units was determined in the dark and also under blue light conditions, a slightly higher activity of about 4,200 Miller units was measured under white light conditions (Figure 5B). When comparing these values with those measured for the wild type and the *ppsR::Tn* mutant, these results pointed toward a repressing function of the PpaA protein under light and blue light conditions, although expression levels measured for the *ppsR*::Tn mutant strain were not reached. For the PpsR regulator it was proposed, that under white and blue light conditions PpsR is acting as a repressor of the *bchFNBHLM* operon. Here, it is striking that even in the dark the *bchF*-*lacZ* expression in the *ppsR*::Tn mutant strain was more that 2-fold higher compared to the expression in the wild type strain (Figure 5B).

### Characterization of the PpsR binding sites within the *bchF* promoter

As described above, four potential PpsR binding motifs have been found bioinformatically within the *bchF* promoter region used for the *bchF-lacZ* reporter gene fusion, sharing the palindromic sequence 5′-TGT-N_12_-ACA-3′ (Figure 5A). Motif 1 (5′-TGT-N_12_-ACA-3′) partly overlaps with the proposed transcriptional start site of *bchF* and the −10 region, while motif 2 (5′-TGT-N_12_-ACA-3′) overlaps with the predicted −35 region (Figure 5A). Therefore, binding of PpsR should prevent binding of the vegetative sigma factor (σ^70^) and the RNA polymerase and as a consequence inhibit transcriptional activation of the operon. We decided to mutate the potential PpsR binding motifs within the *bchF*-*lacZ* reporter gene fusion. Since a mutation of the −35 and − 10 region would impair binding of the RNA polymerase to the *bchF* promoter, only half sites of the palindromic sequences outside of the −10 and −35 region were mutated. The binding motif 1 was mutated to 5′-TGT-N_12_-TTG −3′ and motif 2 to 5′-CCA-N_12_-ACA-3′ resulting in the *bchF*(mut)-*lacZ* reporter gene fusion construct. In the *D. shibae* wild type strain *bchF*(mut)-*lacZ* expression levels of about 9,000 Miller units in the dark, 6,500 Miller units under blue light and 7,800 Miller units under white light conditions were determined (Figure 5B). These values are comparable to the expression levels of the *bchF*-*lacZ* fusion in the *ppsR*::Tn mutant strain. Clearly, these results indicated that the introduced mutations within the *bchF* promoter abolish binding of PpsR and thereby leading to derepression. Thus, the functionality of the proposed PpsR binding sequences were experimentally confirmed.

### Identification of Dshi_1135 interaction partners by interactomics studies

**Figure 6:**
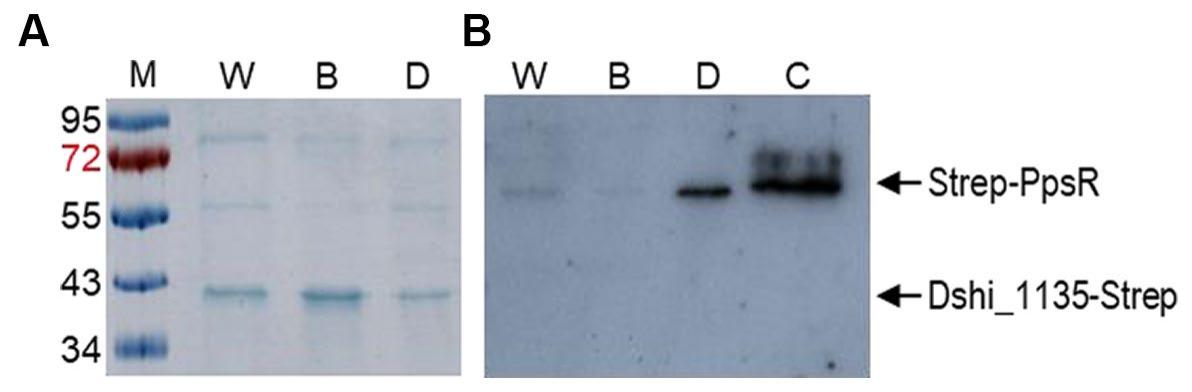
Co-affinity purification identified PpsR as an interaction partner of Dshi_1135. Dshi_1135::Tn was complemented with vector pRhoKS_Dshi_1135-Strep encoding the Dshi_1135-Strep fusion protein under the control of the constitutive *aphII* promoter. After growth under white light (W), blue light (B) and dark (D) growth conditions cells were treated with 0.125% formaldehyde to crosslink protein–protein interaction partners. Dshi_1135-Strep and interaction partners were co-purified via binding on a Streptactin column. Elution fractions were separated on a 15% SDS-PAGE together with a prestained protein marker (M). Separated proteins were stained by Coomassie blue **(A)** and subjected to a western blot for detection of PpsR using a PpsR specific antiserum **(B)**. Purified recombinant Strep-PpsR protein served as a control (C).

The blue light-dependent LOV protein Dshi_1135 showed a clear impact on the transcriptional activation of the PGC. Dshi_1135 is designated an orphan histidine kinase since no response regulator was found associated. Since the histidine residue needed for autophosphorylation of Dshi_1135 is not present and no autophosphorylation was observed an alternative function of Dshi_1135 was assumed. Expression data of the PGC indicated a potential role of Dshi_1135 as an antirepressor of PpsR. This function is usually mediated by a stable protein–protein interaction. In order to prove this hypothesis, co-affinity purification analyses (interactomics) were established to identify the protein interaction partners of Dshi_1135 as successfully described before by our group (Borrero-de Acuña et al., 2015, 2016, 2017). For this purpose, Dshi_1135 was expressed in the Dshi_1135:Tn mutant strain as fusion protein with a C-terminal StrepII-tag. As described above, this complemented strain regained the typical pink pigmentation. Obviously, the produced Dshi_1135-Strep fusion protein is fully functional. The strain was cultivated for approximately 16 h under dark, white light or blue light conditions. Samples were taken to determine the average abundance of soluble proteins by shotgun proteomic analysis using nanoLCESI-iontrap MS/MS (Zech et al., 2013; Wöhlbrand et al., 2017). In parallel, interaction partners of Dshi_1135 were crosslinked by adding formaldehyde *in vivo*. Subsequently, cells were lysed and Dshi_1135-Strep and bound interaction partners were co-purified by affinity chromatography. Proteins in the elution fractions were identified by mass spectrometry (Zech et al., 2013; Wöhlbrand et al., 2017). If a protein was determined in significant larger amounts in the elution fraction of the affinity column in comparison with the natural appearance in the proteomic analysis of the disrupted *D. shibae* strain prior to affinity chromatography purification, its interaction with Dshi_1135 was concluded (Borrero-de Acuña et al., 2015, 2016). As expected, the major protein identified in the elution fraction was the Dshi_1135-Strep bait protein (Table 2). Comparing the peptide counts of the elution fractions with the proteomic data, an enrichment factor of 8 to 10 was calculated for Dshi_1135-Strep. Moreover, in total 260 proteins were identified in the co-elution fraction, but only for 15 proteins a significant enrichment was found for the co-purification. Among those 15 proteins the PpsR repressor was present. It was co-purified under light, blue light and dark conditions while no PpsR protein was detected in any of the proteome fractions (Table 2). Additionally, one must take into account that under dark conditions about 10-fold less Dshi_1135 was purified compared to white light and blue light conditions. Thus, the relative amount of co-purified peptides is higher compared to the blue light/white light conditions. These results were also confirmed by specific detection of PpsR using a PpsR specific antiserum. Proteins of the elution fractions were separated on a 15% SDS-PAGE and either stained by Coomassie blue or subjected to a western blot to detect PpsR. The main protein stained by Coomassie blue was Dshi_1135-Strep with a gene-deduced molecular weight of 38.5 kDa. It was found in comparable amounts under all three light conditions, PpsR was detected mainly under dark conditions (Figure 6). These results clearly indicated a specific interaction of Dshi_1135 with the PpsR repressor and support the role of the blue light-regulated Dshi_1135 protein as an antirepressor mainly under dark conditions. Furthermore, the co-purification attempt did not identify the presence of any cognate response regulator. In addition, the co-purification assay resulted in the enrichment of RpoA, RpoB, RpoD, and RpoC, subunits of the RNA polymerase (Table 2). Moreover, the transcriptional termination protein NusA could be co-purified by binding to the RNA polymerase. These subunits are expected to be in close contact with each other and could therefore be co-purified as a complex together with Dshi_1135 after crosslinking.

**Table 2:** Specific enrichment of interacting proteins by co-affinity purification with the Dshi_1135-Strep protein.

		Proteome			Co-purification		
	Function	White light	Blue light	Dark	White light	Blue light	Dark
Dshi_1135	Blue-light-dependent regulator	5	7	1	229	346	30
PpsR	Transcriptional repressor	nd	nd	nd	7	7	1
RpoA	RNA polymerase subunit alpha	2	3	11	10	6	nd
RpoB	RNA polymerase subunit beta	3	3	9	20	18	4
RpoC	RNA polymerase subunit beta‘	10	7	7	16	16	6
RpoD	RNA polymerase sigma factor	nd	nd	nd	4	13	1
RpoZ	RNA polymerase subunit omega	nd	nd	nd	1	2	nd
NusA	Transcription elongation protein NusA	1	nd	2	4	3	1
GlmU	*N*-acetylglucosamine-1-phosphate uridyltransferase	nd	nd	nd	10	12	nd
Tsf	Elongation factor Ts	2	nd	5	nd	6	1
Eno	Enolase	1	1	5	4	4	nd
Dshi_1084	Conserved hypothetical protein	nd	nd	1	2	3	6
PccA	Propanonyl-CoA carboxylase alpha chain	nd	nd	2	57	52	15
PccB	Propanonyl-CoA carboxylase beta chain	nd	1	1	45	51	18
Dshi_2485	Pyruvate carboxylase	nd	nd	nd	25	22	5
AccB	Acetyl-CoA carboxylase	nd	nd	nd	8	6	nd

Comparison of protein peptide counts of cell extracts and co-elution fractions determined by nanoLC-MS/MS. Median values of three independent experiments are given. nd, not detected.

The carbamoyl-phosphate synthase PccA, the propionyl-CoA carboxylase beta chain PccB, the AccB protein, a component of the acetyl coenzyme A carboxylase complex and protein Dshi_2485 annotated as another pyruvate carboxylase were purified together with the Dshi_1135 protein. Since these proteins are proposed to be biotin utilizing proteins, direct high affinity binding to the streptavidin column can be concluded. The role of *N*-acetylglucosamine-1-phosphate uridyltransferase (GlmU), elongation factor (Tsf), the glycolytic enzyme enolase (ENO) and the protein of unknown function Dshi_1084 remains to be determined (Table 2).

### Interaction studies of Dshi_1135, PpaA, and PpsR using the bacterial two-hybrid system

**Figure 7:**
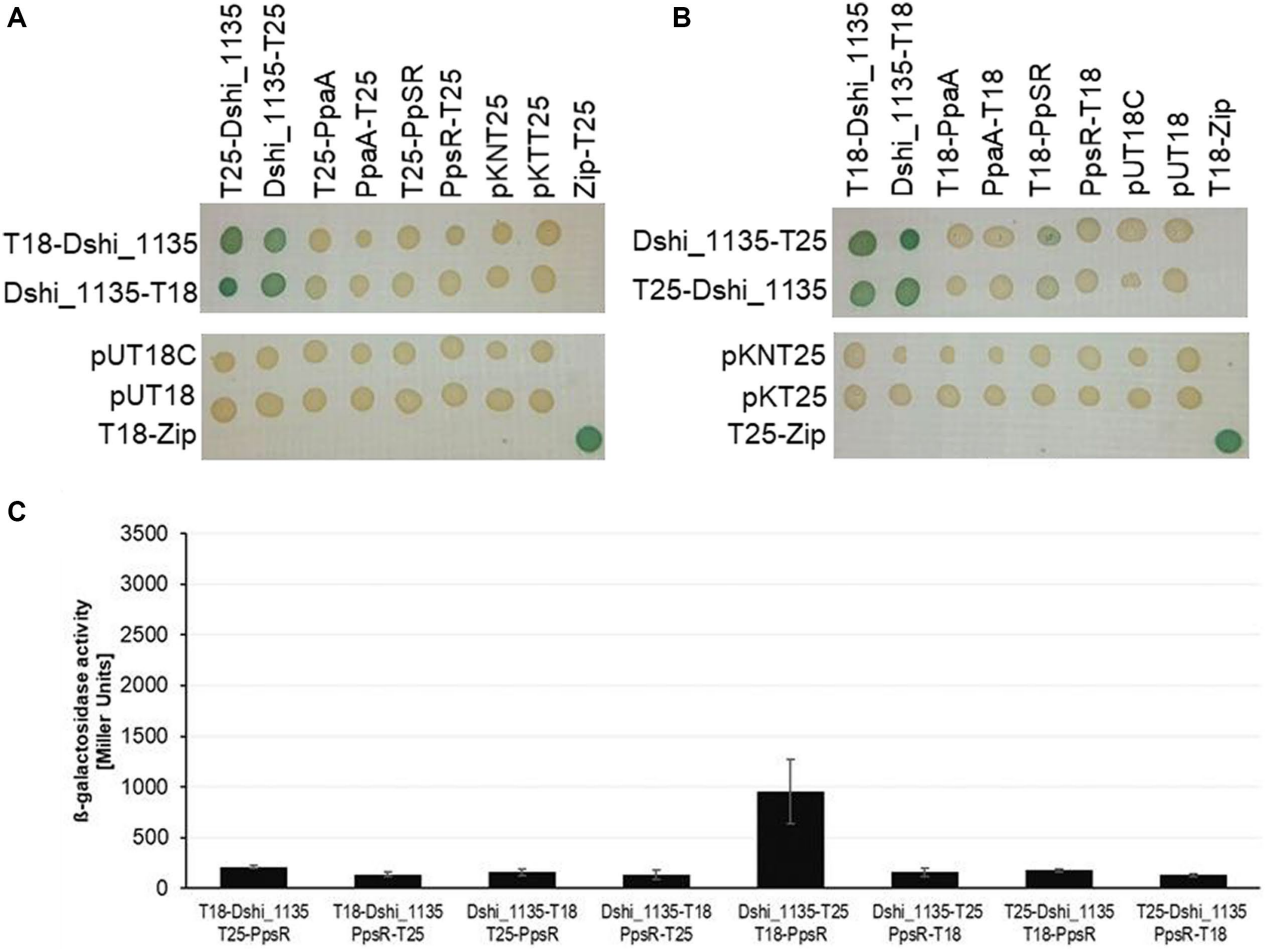
Interaction of Dshi_1135 with PpaA and PpsR tested in the BACTH system. Strain *E. coli* BTH101(Δ*cyaA*) was co-transformed pairwise with **(A)** plasmids encoding C- or N-terminal T18 fusion constructs with Dshi_1135 (Dshi_1135-T18, T18-Dshi_1135) together with C- or N-terminal T25 fusions of Dshi_1135 (Dshi_1135-T25, T25-Dshi_1135) or PpaA (PpaA-T25, T25-PpaA) or PpsR (PpsR-T25, T25-PpsR); or **(B)** with plasmids encoding C- or N-T25 fusion of Dshi_1135 (Dshi_1135-T25, T25-Dshi_1135) together with T18 fusions of Dshi_1135 (Dshi_1135-T18, T18-Dshi_1135) or PpaA (PpaA-T18, T18-PpaA) or PpsR (PpsR-T18, T18-PpsR). Combinations with the empty vectors pKNT25 and pKT25 or pUT18C and pUT18 served as negative controls. The leucine zipper fusion proteins T18-Zip and T25-Zip are applied as positive control (Karimova et al., 2001). Strains harboring the given vector combinations were cultivated overnight in the dark in LB medium containing 0.5 mM IPTG and 2 μL of cell culture was subsequently spotted on LB agar plates containing 0.5 mM IPTG and 40 μg/mL X-Gal. Blue color indicated interaction of the combined proteins. **(C)** β-Galactosidase activities were determined in triplicates from 20 μL of overnight culture under dark growth conditions in 96 well plates and quantified in Miller Units (Miller, 1992).

The adenylate cyclase-based bacterial two hybrid (BACTH) system (Euromedex, Souffelweyersheim, France) was used to investigate the interaction of the Dshi_1135 protein and PpaA protein with PpsR (Karimova et al., 2001). All proteins tested for interaction were expressed with N- and C-terminal T25 and T18 fusions and co-transformed in all combinations in *E. coli* BTH101. First β-galactosidase activities were monitored on LB plates containing X-Gal as a substrate for the β-galactosidase after incubation of the plates in the dark (Figures 7A,B). Blue color indicated interaction of the respective fusion proteins, while all combinations of the fusion proteins with the empty vectors remained colorless as expected (Figures 7A,B). Blue color of the four clones containing all combinations of Dshi_1135 fusion proteins indicated die ability to form dimers or multimers (Figures 7A,B). Combination of the T18-Dshi_1135 or Dshi_1135-T18 fusion protein with any T25 fusion of PpaA or PpsR did not result in blue colonies. No protein–protein interaction could be determined using these combinations (Figure 7A). Once T25-Dshi_1135 or Dshi_1135-T25 was combined with T18 fusions of PpaA or PpsR, a light blue colony only occurred by the combination of Dshi_1135-T25 with T18-PpsR, indicating an interaction of these two regulators (Figure 7B). To quantify the protein-protein interactions, we determined the ß-galactosidase activities of all combinations of Dshi_1135 with PpsR fusion proteins after exposing the cells to dark growth conditions (Figure 7C). The combination of Dshi_1135-T25/T18-PpsR resulted in a β-galactosidase activity of approximately 950 Miller units indicating interaction between Dshi_1135 and PpsR fusion proteins. All other combinations tested showed β-galactosidase activities of about 100 to 200 Miller Units, in the range of the negative controls. Only the combination Dshi_1135-T25/T18-PpsR resulted in a significant interaction. Possibly, the other fusions hinder the interaction of PpsR and Dshi_1135 (Figure 7C).

### Vitamin B_12_-dependent interaction studies of PpaA and PpsR

**Figure 8:**
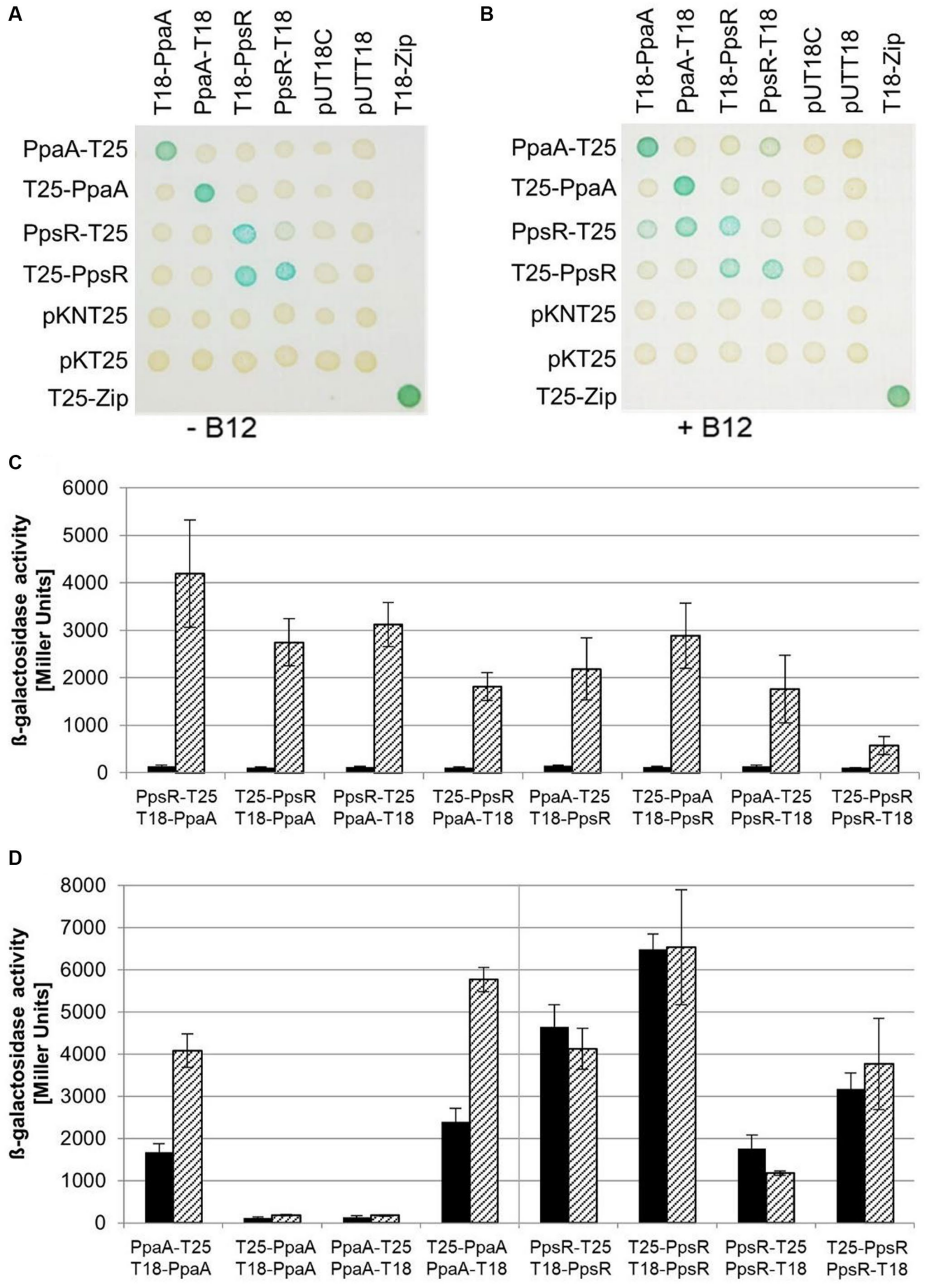
Interaction of PpaA and PpsR tested in the BACTH system. *E. coli* BTH101(Δ*cyaA*) was co-transformed pairwise with plasmids encoding C- or N-terminal T25 fusion constructs with PpaA (PpaA-T25, T25-PpaA) or PpsR (PpsR-T25, T25-PpsR) and with C- or N-terminal T18 fusions of PpaA (T18-PpaA, PpaA-T18) or PpsR (T18-PpsR, PpsR-T18). Combinations with the empty vectors pKNT25 and pKT25 or pUT18C and pUT18 served as negative controls. The leucine zipper fusion proteins T18-Zip and T25-Zip are applied as positive control (Karimova et al., 1998, 2001). Strains harboring the given vector combinations were cultivated overnight in the dark in LB medium containing 0.5 mM IPTG and 2 μL of cell culture was subsequently spotted on LB agar plates containing 0.5 mM IPTG and 40 μg/mL X-Gal in the absence **(A)** or presence **(B)** of 15 nM vitamin B_12_. Blue color indicated interaction of the combined proteins. **(C,D)** β-Galactosidase activities were determined from 20 μL of overnight culture in 96 well plates and quantified in Miller Units (Miller, 1992). ß-Galactosidase activities derived from cultures grown in the absence (black bars) or presence (dashed bars) of 15 nM hydroxy cobalamin.

The interaction of the proposed antirepressor PpaA and PpsR was then analyzed using the BACTH system on agar plates containing X-Gal as a substrate for β-galactosidase activity. Indicated by the blue coloration we determined that the PpaA protein fusions were able to form dimers or multimers, but only if a N-terminal PpaA fusion was combined with a C-terminal fusion protein (PpaA-T25/T18-PpaA and T25-PpaA/PpaA-T18). Interaction was abolished when both adenylate cyclase domains were either fused N- or C-terminally (Figure 8A). In contrast, the PpsR protein fusions formed dimers or multimers in any combination tested (Figure 8A). Surprisingly, no β-galactosidase activity was detected by combining PpaA fusion proteins in any combination with PpsR fusion proteins. Since PpaA of *C. sphaeroides* and AerR of *R. capsulatus* are supposed to activate transcription of the PGC by acting as an antirepressor of PpsR, we expected an interaction signal for PpaA and PpsR fusion proteins in this assay. As mentioned above, the PpaA protein family members exhibit amino acid sequence similarity to a previously defined SCHIC (sensor containing heme instead of cobalamin) domain and it was shown, that PpaA is binding hydroxy cobalamin (Vermeulen and Bauer, 2015). To prove whether binding of vitamin B_12_ is crucial for interaction of PpaA and PpsR fusion proteins, we added 15 nM hydroxy cobalamin to the growth medium of the agar plates. Addition of hydroxy cobalamin led to blue colored spots while combining PpsR-T25 protein fusion with T18-PpaA as well as PpaA-T18. Also, the combination of PpaA-T25 with T18-PpsR or PpsR-T18 resulted in blue colored spots (Figure 8B). To quantify this B_12_-mediated interaction of PpaA and PpsR fusion proteins, we determined the β-galactosidase activities derived from all eight different combinations of PpaA and PpsR fusion proteins (Figure 8C). When the cells were cultivated in the absence of hydroxy-cobalamin about 100 Miller units were obtained, whereas in the presence of 15 nM hydroxy cobalamin about 570 Miller units were obtained by interaction of PpsR-T18 with T25-PpaA. The highest yield of about 4,200 Miller units resulted from the combination of the T18-PpaA fusion protein with PpsR-T25. Again, values of β-galactosidase activities changed depending on the location of the fused T25 or T18 domain to the regulator proteins. But it is a fact that the addition of B_12_ allows PpaA to interact with PpsR, probably by changing the conformation of the antirepressor. In contrast, multimerization of PpaA fusion proteins was affected by addition of B_12_. Here, β-galactosidase activity was doubled indicating that the cofactor would either stabilize the corresponding fusion proteins or promote oligomerization In contrast, oligomerization of PpsR fusion proteins was totally unaffected by B_12_ in the BACTH assay (Figure 8D).

### *In vivo* antirepressor function of Dshi_1135

**Figure 9:**
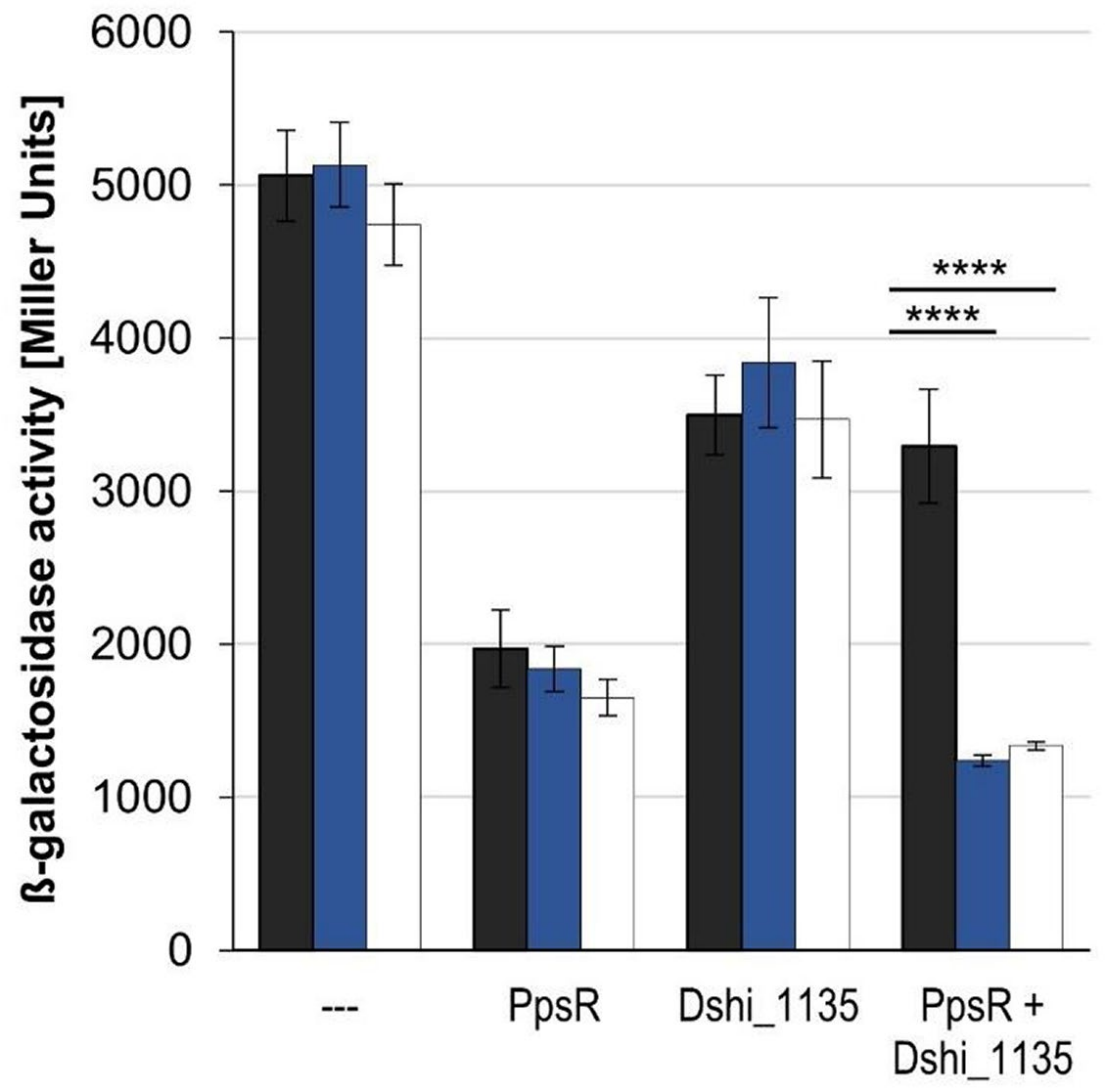
*In vivo* antirepressor function of Dshi_1135 is light-dependent. The expression of the *bchF-lacZ* reporter gene fusion derived from plasmid pACYC_*bchF-lacZ* was studied in *E. coli* Novablue cells (EMD Biosciences, San Diego, United States) dependent of the presence of the PpsR protein or Dshi_1135 protein production alone or in combination of both proteins after cultivation in the dark (black bars), under blue light illumination (blue bars) or white light conditions (white bars). Protein production was derived from the vector pETDuet-1 harboring *ppsR* or Dshi_1135 alone or both together and induced by addition of 10 μM IPTG. The empty vector pETDuet-1 served as control. ß-Galactosidase activities were determined in the mid-log growth phase after 17 h of cultivation. Experiments were repeated three times with similar results. Mean values of the β-galactosidase activities and the corresponding standard deviations are indicated in Miller Units. *p*-values were determined by two-way ANOVA and Tukey’s *post hoc* test (^****^p ≤ 0.0001).

In order to analyze the proposed antirepressor function of Dshi_1135, a heterologous expression system in *E. coli* was established. The idea was to study the *D. shibae* Dshi_1135—*D. shibae* PpsR interaction and role for *bchF* gene expression independent from the complex *D. shibae* gene regulatory background including multiple other regulatory proteins to obtain clear-cut results. Transcriptional repression and derepression was monitored using the *bchF-lacZ* reporter gene fusion, which is highly expressed in *E. coli* with a β-galactosidase activity of approximately 4,000 to 5,000 Miller Units, independent of cultivation in the dark, light or under blue light illumination (Figure 9). In addition, *ppsR* and Dshi_1135 genes were expressed solely or in combination from the IPTG inducible promotor of the co-transformed vector pETDuet-1. Production of PpsR alone resulted in ß-galactosidase activities in a range of approximately 1,000 to 2,000 Miller units, indicating a substantial repression of *bchF-lacZ* expression under all light conditions tested (Figure 9). Production of Dshi_1135 alone led to a slight decreased expression compared to the *bchF-lacZ* reporter gene alone in the range of about 3,500 to 3,800 Miller units. Combined recombinant production of PpsR and Dshi_1135 under dark conditions resulted in β-galactosidase activities of about 3,200 Miller units and restored almost full expression of *bchF-lacZ* clearly indicating antirepressor function of Dshi_1135. In contrast, the same strain cultivated under white and blue light conditions only resulted in approximately 1,200 to 1,300 Miller units in a range also measured when PpsR was produced without Dshi_1135. These results again clearly indicate an antirepressor function of Dshi_1135, only under dark growth conditions.

## Discussion

**Figure 10:**
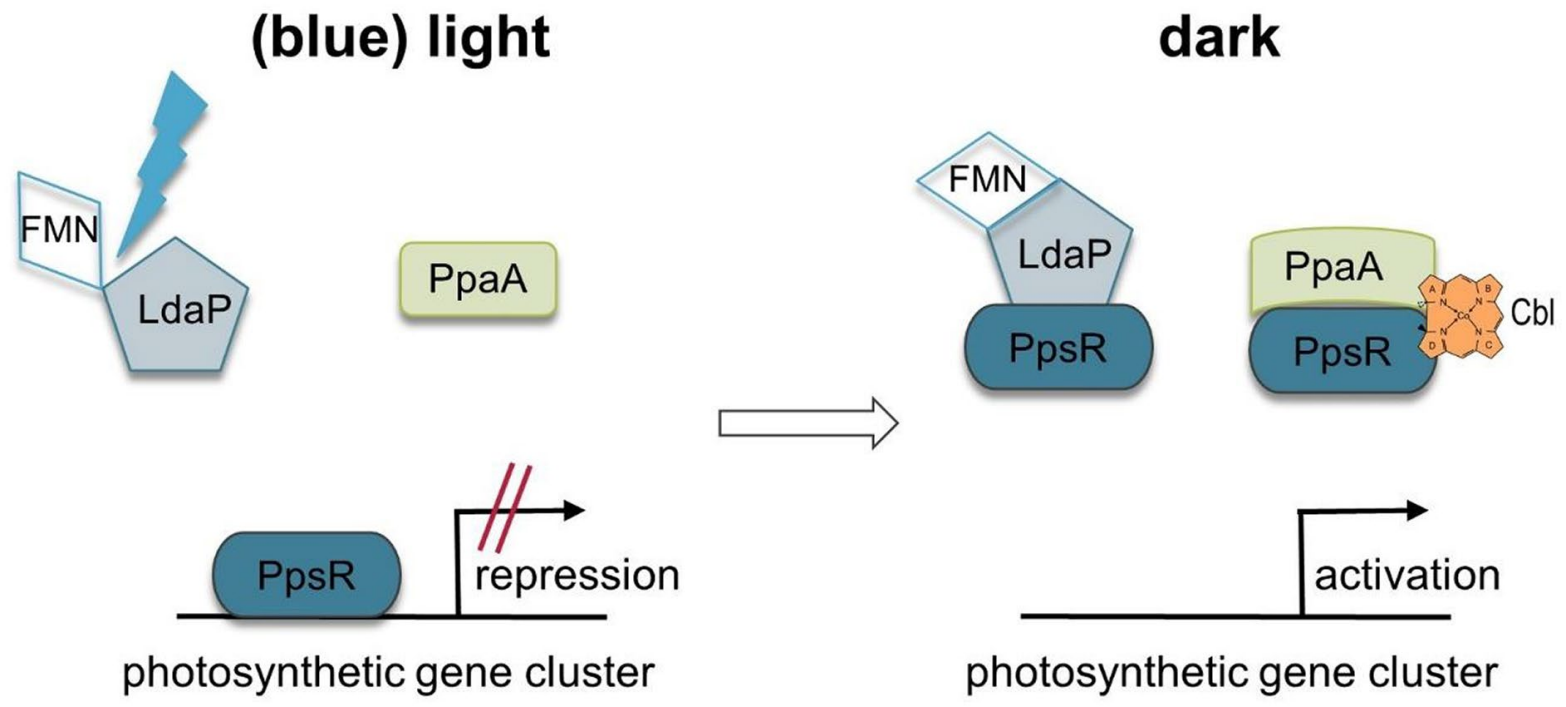
Model of light-dependent regulation of the photosynthetic gene cluster in *Dinoroseobacter shibae.* Under blue light and light conditions PpsR is binding as a repressor to the promoter region and inhibits transcriptional activation. The blue light sensing Dshi_1135 protein is in its inactive form. Under dark light conditions Dshi_1135 is able to interact with PpsR which in turn is no longer able to bind to the DNA. Subsequently, transcriptional activation is possible. In addition, antirepressor PpaA is able to bind PpsR in the presence of hydroxy cobalamin and supports transcriptional activation.

This manuscript details the successful screening of the transposon library in *D. shibae* to identify regulatory proteins implicated in the light-dependent expression of the photosynthetic gene cluster. The study highlights the discovery of Dshi_1135 as a new antirepressor of PpsR, specifically responsive to blue light. The findings are presented in a regulatory model (Figure 10). In this model, PpsR inhibits the transcription of the photosynthetic gene cluster under both white and blue light conditions by binding to at least two conserved sequences (5′-TGT-N12-ACA-3′). These sequences partially overlap with the −10 and −35 promoter regions, thereby preventing RNA polymerase binding (Figure 10). Under light or blue light conditions Dshi_1135 is in its inactive form and not able to bind to PpsR. Transcriptional activation under dark growth conditions is mediated by interaction of the Dshi_1135 protein in the dark state with PpsR preventing repressor function. According to this function protein Dshi_1135 was named LdaP, for light-dependent antirepressor of PpsR. In addition, PpaA is able to interact with PpsR in the presence of cobalamin and act as a second antirepressor of PpsR. *Dinoroseobacter shibae* shares the set of photosynthetic regulators of the CrtJ/PpsR and AerR/PpaA families with *R. capsulatus* and *C. sphaeroides.* Unlike the repressor proteins CrtJ and PpsR of *R. capsulatus* and *C. sphaeroides*, respectively, the PpsR repressor function in *D. shibae* is independent of oxygen, but light-dependent.

*In vivo* ChIP-seq experiments showed that direct interaction between the vitamin B_12_ binding photoreceptor AerR with CrtJ modulates CrtJ binding to DNA. AerR functions as a switch that alters CrtJ binding at target sequences and relieves repressor activity (Fang and Bauer, 2017), indicating that antirepressor function is not simply release of the repressor from the DNA. For PpaA of *C. sphaeroides* specific binding of hydroxy cobalamin and interaction of PpaA and PpsR was already shown (Vermeulen and Bauer, 2015). The PpaA homolog AerR of *R. capsulatus* exists in two isoforms that differ by 41 amino acids at the amino terminus. The larger version of AerR binds vitamin B_12_ only in the presence of light and stimulates CrtJ to activate genes involved in photosynthesis. The shorter version binds to vitamin B_12_ in the dark and causes CrtJ to repress expression of PGC (Cheng et al., 2014; Fang and Bauer, 2017; Yamamoto et al., 2018). Vitamin B_12_ can also function as a chromophore for light absorption to facilitate an output response that affects gene expression as shown for CarH and AerR (Ortiz-Guerrero et al., 2011; Cheng et al., 2016). One could assume, that binding of cobalamin to PpaA in *D. shibae* might contribute to light-dependent regulation of PpsR, but it will not explain derepression of the PGC under dark conditions. Moreover, the *ppaA*::Tn mutant strain still gets pink on ASM and produces photosynthetic pigments, while mutation of the Dshi_1135 gene, encoding the new light-dependent antirepressor LdaP, resulted in a fully white phenotype with total loss of pigmentation. In addition to PpaA, *C. sphaeroides* possesses a blue light photoreceptor, named AppA, that acts as an antirepressor of PpsR during expression of PGC (Gomelsky and Kaplan, 1997; Gomelsky and Kaplan, 1998; Masuda and Bauer, 2002). AppA possesses a N-terminal BLUF domain sensing blue light via a FAD cofactor. Corresponding light-induced structural changes of the apoprotein lead to association with the transcriptional repressor PpsR (Gomelsky and Klug, 2002; Masuda et al., 2005). Structural analyses determined a ternary AppA–PpsR–DNA complex that mediates light-regulation of photosynthesis-related gene expression (Winkler et al., 2013). Neither *R. capsulatus* nor *D. shibae* possesses an AppA homolog. In contrast, *D. shibae* harbors LdaP, a LOV-dependent blue light sensing antirepressor of PpsR.

## Materials and methods

### Bacterial strains and growth conditions

The type strain *Dinoroseobacter shibae* DFL12^T^ served as wild type strain (Biebl et al., 2005). *Dinoroseobacter shibae* transposon mutant strains were derived from a transposon library described before (Ebert et al., 2013). The integration sites were identified by arbitrary PCR as described in Ebert et al. (2013). All strains were grown aerobically at 30°C and 200 rpm in artificial seawater medium (ASM) with 16.9 mM succinate (Tomasch et al., 2011) or Marine Bouillon (MB; Roth, Karlsruhe, Germany). Dark conditions were obtained by fully covering the culture flasks (aquila biolabs, Baesweiler, Germany) or using a dark covered cabinet. For incubation at different light conditions we used a temperature controlled cabinet containing three types of LED strips emitting light at 467, 515, and 628 nm, respectively (type 18418 1-3, Barthelme, Germany), which was already used in a previous work of our group (Kirchhoff et al., 2018). The intensity was adjusted to 9.6 μmol photons m-2 s-1 using a dimmer (light meter model LI-189 from LI-COR, United States). For white light, all three LED types were activated simultaneously, each with an intensity of 9.6 μmol photons m-2 s-1. *E. coli* strains were grown in Lysogeny Broth (LB) supplemented with appropriate antibiotics and amino acids at 37°C and 200 rpm, if not indicated otherwise.

### Magnesium protoporphyrin IX monomethyl ester accumulation

Magnesium protoporphyrin IX monomethyl ester (MPE) accumulation by the tested *D. shibae* transposon mutant strains was observed on ASM agar plates solidified with 1.5% agar. After incubation for 24 h in MB 5 μL of the culture were dropped on the ASM agar plates and incubated for 48 h at 30°C. The fluorescence of the resulting colonies was monitored using a blue light transilluminator (Flu-O-Blu; Biozym, Hessisch Oldendorf, Germany).

### Bacteriochlorophyll extraction and UV/vis spectroscopy

For pigment extraction the amount of cells corresponding to an OD_578_ = 2.5 of the analyzed *D. shibae* strains were sedimented by centrifugation (10 min, 2,500 × g, 4°C). The supernatant was discarded and the pellet was dissolved in 1 mL acetone/methanol (7:2) solution. Pigment extraction was done on a tumbler mixer at room temperature for 1 h in the dark. Cell debris was removed by centrifugation for 5 min at 2,500 × g and the resulting supernatant was transferred into a quartz cuvette for UV/vis spectroscopy. The spectra were recorded in a range of 300–900 nm on a V-550 spectrometer (Jasco, Gross Umstadt, Germany). The acetone/methanol (7:2) solution served as blank (Gough et al., 2007).

### Transcriptome analysis

For transcriptomic analyses *D. shibae* strains were cultivated in dark in ASM as described above. Samples were taken at an OD_578_ of 0.5 and RNA was extracted by using RNAprotect solution and the RNeasy Mini Kit according to manufacturer protocols (Qiagen, Hilden, Germany). One μg of total RNA was labeled with Cy3 using the ULS-system (Kreatech, Amsterdam, Netherlands) according to the manufacturer’s manual. The labeled RNA (600 ng) was fragmented and hybridized to the custom designed gene specific DNA microarray (Agilent, Santa Clara, United States 8 × 15 K format) according to Agilent’s one-color microarray protocol. Arrays were scanned on the Agilent C scanner. Data evaluation was performed in the R environment using the following packages: limma, Biobase, gplots, marray, affy, combinate and graphics.^2^ Microarray analyses were performed with four biological replicates. Only genes with a logarithmic change of ≥0.8 comparing expression levels of wild type and mutant strains with a *p*-value < 0.05 were considered for subsequent analyses. Generated data have been deposited in NCBI’s Gene Expression Omnibus (Edgar et al., 2002; Barrett et al., 2013) and are accessible through Geo Series accession number GSE244289.

### Cloning, expression and purification of Dshi_1135

The DNA encoding sequence of the potential LOV protein Dshi_1135 (GenBank: ABV92877.1) was PCR amplified from *D. shibae* DFL12^T^ genomic DNA, using the primer pair EH649 (5′-GGGATGAAGCCACGCACGCTAGGA-3′) and EH650 (5′ GCGAGCTCAGCCATCTGATTGCGGTC 3′). The PCR product was purified using the Qiagen PCR purification kit and subsequently cloned into the pET52b(+)Trx vector, a derivative of vector pET52b(+) (Frädrich et al., 2012). Cloning via the restriction sites *Sma*I and *Sac*I resulted in plasmid pET52b(+)Trx_Dshi1135, which was transformed into *E. coli* BL21 (DE3) CodonPlus cells for production of the Trx/StrepII-Dshi_1135 fusion protein. Cells were grown in 500 mL Lysogeny broth (LB) supplemented with 100 μg/mL ampicillin at 37°C to an OD_578_ of 0.5–0.6 and production was induced by addition of 50 μM isopropyl 1-thio-β-D-galactopyranoside (IPTG). Immediately after IPTG addition cultures were shifted to 17°C for further incubation and after 18 h the cells were harvested by centrifugation. The resulting cell pellet was resuspended in 20 mL 50 mM Tris–HCl (pH 8.2) and 500 mM NaCl buffer and subsequently disrupted using a French press at 19,200 psi. The soluble protein fraction was obtained by ultracentrifugation at 121,000 × g at 4°C for 65 min and subsequently loaded onto 1 mL of *Strep-*Tactin Superflow high-capacity resin (IBA, Göttingen, Germany), equilibrated with washing buffer (50 mM Tris–HCl (pH 8.2), 150 mM NaCl, 5 mM MgCl_2_, 10% (w/v) glycerol). After extensive washing with 20 column volumes of washing buffer, bound proteins were eluted using washing buffer supplemented with 2.5 mM *d*-desthiobiotin. All protein containing fractions were analyzed by SDS-PAGE and protein concentrations were determined using Bradford reagent (Sigma-Aldrich, St. Louis, MO, United States) according to manufactures instructions. Due to the light sensitivity of Dshi_1135, all purification steps were performed under dim red-light conditions. For reconstitution, purified Dshi_1135 protein was mixed well with 100 μM FMN and the solution was incubated for 30 min at 4°C under constant rolling. To remove unbound FMN the protein-FMN mixture was loaded onto a NAP-25 Column (GE Healthcare, Munich, Germany) prepared according to manufacturer’s instructions and the protein was eluted in washing buffer. Reconstituted Dshi_1135 protein was stored at 4°C under dark conditions.

### Site-directed mutagenesis

The plasmid pET52b(+)_Trx/StrepII-Dshi1135C61A for the recombinant production of the mutant protein Dshi_1135C61A was generated using the Q5 Site-Directed Mutagenesis Kit (New England Biolabs, Frankfurt am Main, Germany) according to manufacturer’s instructions using the primer pair MIB14 (5′-CGGCCGGAACGCGCGGTTCCTGCAAGGCAGC-3′)/MIB15 (5′-ATCGCCGCGGTGCGCGAA-3′) and plasmid pET52b(+)Trx_Dshi1135 as a template.

### Autophosphorylation assay

Protein concentrations between 10 and 40 μM, [γ-^32^P] ATP concentrations between 5 and 10 μCi (Hartmann Analytic, Braunschweig, Germany) and non-labeled ATP concentration from 1 μM to 1 mM were tested. Sample collections occurred 0.5, 1, 2, 4, 8, 16, 30, 60 min and 24 h after reaction start and incubation at room temperature under dim red light conditions or exposed to blue light (467 nm; LED growth light, LL-GL001, Albrillo). At desired time points samples were quenched by adding 2× SDS loading dye and subsequent flash freezing in liquid nitrogen. Samples were denatured for 10 min at 95°C and separated on a 12% (w/v) SDS gel. The gel was then transferred to a Whatman paper and exposed overnight to a X-ray film (Fuji Medical X-Ray Film Super RX-N, Fujifilm, Minato, Tokyo, Japan) at room temperature.

### Phos-tag™ SDS-PAGE

4–15 μM of the purified protein were mixed with ATP (1 μM, 10 μM, 100 μM, and 1 mM) under red light conditions at room temperature and incubated for 24 h either in the dark or under blue light (467 nm; LED growth light, LL-GL001, Albrillo) irradiation. After incubation the reaction was stopped by the addition of 2× SDS loading dye and the samples were separated on 10% SDS gels supplemented with 20–100 μM Phos-tag™ and 40–200 μM MnCl_2_ prepared according to manufacturer’s instructions (FUJIFILM Wako Chemicals Europe, Neuss, Germany).

### Ultraviolet–visible absorption spectroscopy

UV/vis spectra from 200 to 700 nm of the purified Dshi_1135 protein were recorded at room temperature in washing buffer containing 2.5 mM *d*-desthiobiotin on a V-550 spectrometer (Jasco, Gross Umstadt, Germany) and a quartz cuvette with 10 mm path length. All protein samples were kept in the dark until spectra measurement. For the recording of spectra of purified Dshi_1135 in the signaling state, the sample was illuminated with blue light (457 nm) for 5 min before data collection.

### Promoter-*lacZ* reporter gene fusions

The pBBRLIC-*lacZ* vector was used for ligation independent cloning of promoter-*lacZ* reporter gene fusions (Ebert et al., 2017). A 329 bp fragment of the *bchF* promoter region, encoding sequences from −289 to +41 with respect to the translation start of *bchF* was PCR amplified from *D. shibae* DFL12^T^ genomic DNA using the primer pair EH677 (5′-CCGCGGGCTTTCCCAGCATGGGATCTTGCAGGTT-3′) and EH678 (5′-GTTCCTCCTTCCCACCAGTCCGACCCTGTTTTCT-3′). Subsequently, the *bchF* promoter fragment was cloned into pBBRLIC-*lacZ*, resulting in the plasmid pBBR1LIC_*bchF*-*lacZ*. For the generation of *bchF*(mut)-*lacZ* reporter gene fusion, the *bchF* promoter fragment containing mutations of two potential PpsR binding sites at positions −79/−77 from TGT to CCA and −30/−28 from ACA to TGG with respect to the translational start site was ordered as synthetic DNA (Thermo Fisher Scientific Inc., Waltham, United States) and subsequently cloned into the pBBRLIC-*lacZ* vector via ligation independent cloning. This resulted in the plasmid pBBR1LIC_*bchF*(mut)-*lacZ.*

### Strain construction for homologous production of Dshi_1135

For homologous co-purification of Dshi_1135-Strep protein interaction partners the *D. shibae* strain DS266 was used. The Dshi_1135::Tn transposon mutant strain (DSTn12510) was complemented by the plasmid pRhoKS_Dshi1135-Strep encoding the Dshi_1135 protein with a C-terminal StrepII-tag under the control of the constitutive *aphII* promoter (Katzke et al., 2010). For plasmid construction the Dshi_1135 gene sequence was amplified using the pET52b(+)Trx_Dshi1135 plasmid as template and primers EH725 (5′-GGAAATTCCATATGAAGCCACGCACGCTAGGA-3′) and MIB29 (5′-CCGCTCGAGATTGCCCGCGGGCAC-3′). The resulting 1,016 bp DNA fragment was ligated into vector pRhokS both digested with *Nde*I and *Xho*I (New England BioLabs, Ipswich, MA, USA) yielding the plasmid pRhokS_Dshi1135-Strep.

### Co-affinity purification

Strain DS266 was grown in 500 mL MB medium for approximately 24 h at 30°C until reaching an OD578 of 0.8 either in the dark, under blue light (467 nm) or white light conditions, respectively. To crosslink the proteins *in vivo* formaldehyde to a final concentration of 0.125% (vol/vol) was added and the bacteria were further incubated for 20 min at 30°C under continuous shaking. Subsequently, free aldehyde groups were quenched by addition of 135 mM glycine and incubation was continued for 5 min at 30°C. Next, the bacteria were harvested by centrifugation (11,300 × *g* for 20 min), the pellet was washed in 50 mM Tris–HCl buffer (pH 8.2) and 500 mM NaCl and finally resuspended in 6 mL of the same buffer and 1 μL of benzonase (25 U/μL; Merck, Darmstadt, Germany) was added. For Strep-Tactin purification of the crosslinked Dshi_1135 protein complexes, cells were disrupted using a French Press (19,200 psi). The cell homogenates were clarified by centrifugation (121,000 × *g* for 60 min at 4°C) and the supernatant was subjected to Strep-Tactin column chromatography. Elution fractions were further analyzed by nanoLC-MS/MS, SDS-PAGE, and Western blotting. Western blots were probed with StrepMAB-Classic HRP (IBA Lifesciences, Göttingen, Germany) and polyclonal anti-Dshi_1135 antiserum (Davids, Regensburg, Germany).

### Protein identification by nanoLC ESI-iontrap MS/MS

For shotgun proteomic analyses, cells were disrupted by using French press (19,200 psi) and the cell homogenates were clarified by centrifugation (121,000 × *g* for 60 min at 4°C). Subsequently, 50 μg total protein were subjected to reduction, alkylation and tryptic in-solution digestas described (Wöhlbrand et al., 2017). Finally, 2.5 μg of the obtained peptide solution were separated using a nano LC (Ultimate 3,000 nanoRSLC; Thermofisher Scientific, Dreieich, Germany) applying a trap column setup (2 cm length, 5 μm bead size, 75 μm inner diameter; Thermofisher Scientific) with a 25 cm separation column (2 μm bead size, 75 μm inner diameter; Thermofisher Scientific) and a 280 min linear gradient. The eluent was continuously ionized (captive spray ion source; Bruker Daltonik GmbH, Bremen, Germany) and ions analyzed by an ion-trap mass spectrometer (amaZon speed ETD; Bruker Daltonik GmbH) as described (Zech et al., 2013; Wöhlbrand et al., 2017). For the Dshi_1135 co-purification samples, a total of 4 μg protein were used for in-solution digest and peptide separation (load 0.3 μg) achieved by a 130 min linear gradient. In all cases, three biological replicate samples per condition were prepared. Protein identification was performed using Mascot (version 2.3; Matrix Science, London, UK) via the ProteinScape platform (version 4.2; Bruker Daltonik GmbH) and a genomic database of *D. shibae*. A target-decoy strategy with a false discovery rate < 1.0% was applied as well as the following settings: enzyme trypsin; one missed cleavage allowed; carbamidomethylation (C) as fixed, oxidation (M) as variable modification; peptide and MS/MS mass tolerance 0.3 Da; monoisotopic; peptide charge 2+ and 3+; instrument type ESI-TRAP; significance threshold *p* < 0.05; ion score cutoff 25.0; minimum peptide length of five.

### Bacterial two hybrid plasmids construction and interaction studies

Bacterial adenylate cyclase two hybrid (BACTH) assays were carried out with the non-reverting adenylate cyclase-deficient (*cya^−^*) *E. coli* strain BTH101 (Karimova et al., 1998; Battesti and Bouveret, 2012). For the construction of recombinant plasmids used for BACTH assays, the genes of interest were cloned in frame with either the T18 or the T25 fragment of the catalytic domain of the *Bordetella pertussis* adenylate cyclase (*cyaA*) into the vectors pUT18C, pUT18, pKNT25 and pKT25 of the BACTH system kit (Euromedex, Souffelweyersheim, France). Cloning was conducted using the HiFi DNA assembly protocol (NEB, Ipswich, Massachusetts, USA) and primers listed in Supplementary Table S1. Given pairs of these plasmids were introduced into *E. coli* strain BTH101(*cya*^−^; Supplementary Table S2). Interaction was assessed qualitatively from the blue color developed on LB plates containing X-Gal, and quantitatively by the β-galactosidase specific activity measurements in at least three independent experiments for each interacting pair tested. The transformants were plated onto LB/X-Gal/IPTG (40 μg/mL 5-bromo-4-chloro-3-indolyl-D-galactopyranoside, 0.5 mM IPTG) agar plates with ampicillin (100 μg/mL) and kanamycin (50 μg/mL). Negative controls consisted of pairs with only one fusion protein expressed in combination with the empty pUT18C, pUT18, pKNT25 or pKT25 vector, respectively. In addition, the plasmids pKT25-zip and pUT18C-zip were used as positive controls for BACTH complementation (Karimova et al., 2000). For β-galactosidase activity assays transformants cells were grown for 16 h in 200 μL LB medium at 30°C and 300 rpm in 96 well plates. OD_578_ was determined and 20 μL of the bacterial culture was mixed with 80 μL freshly prepared permeabilization solution (100 mM Na_2_HPO_4_, 20 mM KCl, 2 mM MgSO_4_, 0.6 μg/mL hexadecyltrimethylammonium bromide (CTAB), 0.4 μg/mL sodium deoxycholate, and 5.4 μL/mL β-mercaptoethanol). After at least 30 min incubation the β-galactosidase assay was started by adding 25 μL of the permeabilized bacterial cells to 150 μL freshly prepared substrate solution (60 mM Na_2_HPO_4_, 40 mM NaH_2_PO_4_, 1 mg/mL *o*-nitrophenyl-β-D-galactopyranoside (ONPG), and 2.7 μL/mL β-mercaptoethanol) in a 96 well plate. The OD_420_ and OD_550_ was determined after 30 min in the TECAN reader and β-galactosidase activities were calculated (Miller, 1972).

### β-galactosidase assay

For β-galactosidase assays, *D. shibae* cells were grown in ASM under white light, blue light (467 nm) or dark conditions at 25°C and 200 rpm. Cells were harvested in the mid-exponential growth phase and used for β-galactosidase assays as described previously (Miller, 1992; Härtig et al., 2004).

## Data availability statement

The datasets presented in this study can be found in online repositories. The names of the repository/repositories and accession number(s) can be found at: https://www.ncbi.nlm.nih.gov/geo/, GSE244289.

## Author contributions

SP: Conceptualization, Writing – original draft, Writing – review & editing, Data curation, Investigation, Methodology. MB: Conceptualization, Writing – original draft, Investigation, Methodology. SH: Writing – original draft, Data curation, Investigation, Methodology. LW: Conceptualization, Data curation, Investigation, Methodology, Writing – review & editing, Writing – original draft. RR: Data curation, Formal Analysis, Supervision, Writing – review & editing. DJ: Conceptualization, Funding acquisition, Supervision, Writing – original draft, Writing – review & editing. EH: Conceptualization, Funding acquisition, Project administration, Supervision, Writing – original draft, Writing – review & editing.

## Footnotes

^1^www.prodoric.de

^2^https://www.bioconductor.org
