# Supplementary material for: The blue light-dependent LOV-protein LdaP of Dinoroseobacter shibae acts as antirepressor of the PpsR repressor, regulating photosynthetic gene cluster expression
Source: Front Microbiol. 2024 Feb 7;15:1351297. doi: 10.3389/fmicb.2024.1351297 (PMC10890935; doi:10.3389/fmicb.2024.1351297)
Supplement: Supplementary file 1 [file Data_Sheet_1.docx]

Supplementary Material

The blue light-dependent LOV-protein LdaP from *Dinoroseobacter shibae* acts as an antirepressor of the PpsR repressor, regulating photosynthetic gene cluster expression

**Saskia Pucelik^1^, Miriam Becker^1^, Steffi Heyber^1^, Lars Wöhlbrand^2^, Ralf Rabus^2^, Dieter Jahn^3^ and Elisabeth Härtig^1^***

^1^Institute of Microbiology, Technische Universität Braunschweig, Spielmannstraße 7, D-38106 Braunschweig, Germany

^2^ Institute of Chemistry and Biology of the Marine Environment (ICBM), Carl von Ossietzky University of Oldenburg, Carl-von-Ossietzky-Str. 9-11, D-26111 Oldenburg, Germany

^3^Braunschweig Integrated Centre of Systems Biology (BRICS), Technische Universität Braunschweig, Rebenring 56, D-38106 Braunschweig, Germany

**Fig. S1**

**
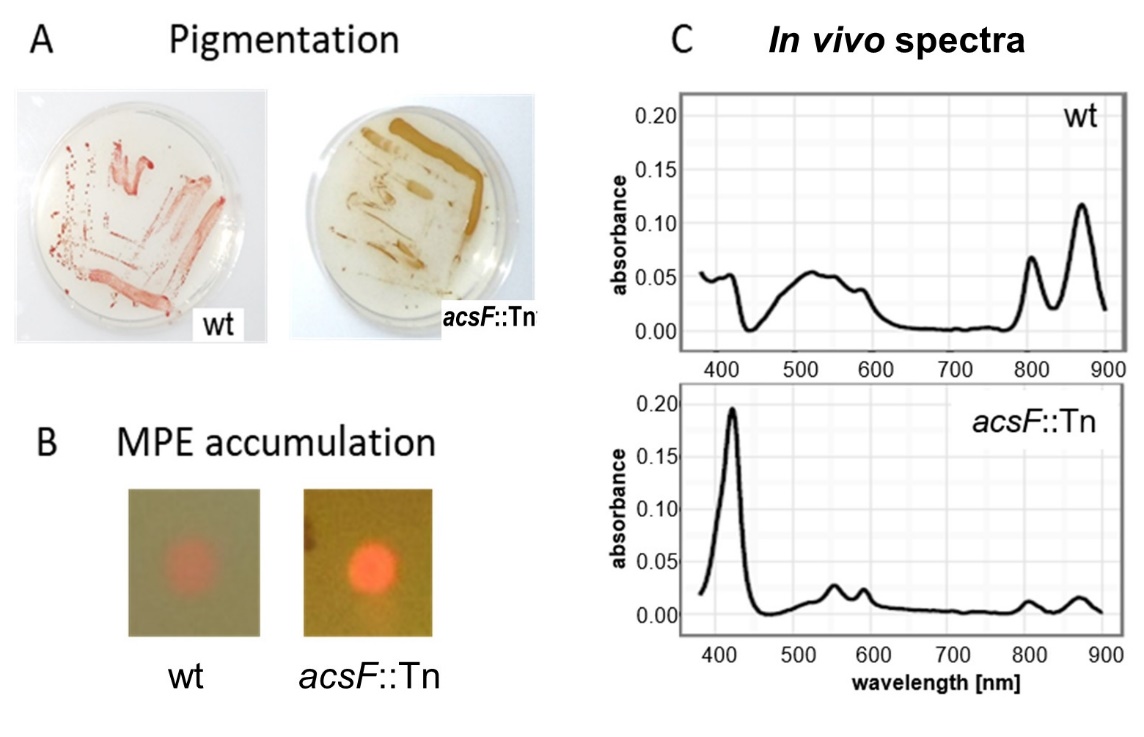
**

**Figure S1. Screening for mutants defective in bacteriochlorophyll biosynthesis. (A)** *D. shibae* transposon library was screened for changes in pigmentation. Shown are the *D*. *shibae* wildtype strain in comparison to the *acsF*::Tn transposon mutant strain. **(B)** Accumulation of magnesium-protoporphyrin IX monomethyl ester (MPE), a fluorescent intermediate of bacteriochlorophyll biosynthesis with an absorption maximum at 416 nm in the the *acsF*::Tn transposon mutant. **(C)** The *in vivo* absorption spectrum of the *D. shibae* wildtype strain (wt) mainly reflects the combination of bacteriochlorophyll absorption at 374 nm (Soret), 590 nm (Qx) and 804 and 868 nm (Q_y_) and the carotenoid absorption at 450 to 570 nm. The absorption spectrum was compared to the *in vivo* spectrum of the *acsF*::Tn mutant strain. The major peak resulted from MPE absorption at 416 nm.

**Fig. S2**


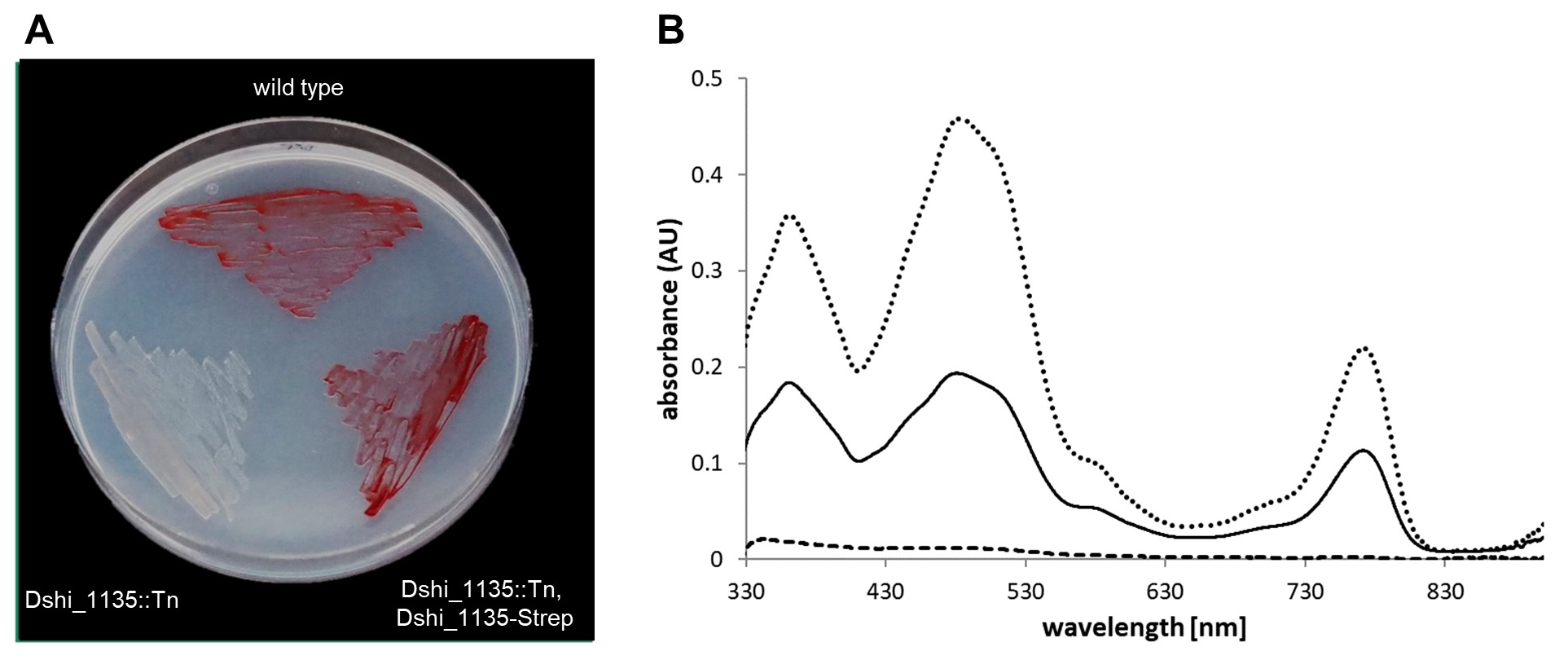


**Figure S2. Komplementation of the Dshi_1135::Tn Mutant strain fully restore pigmentation to wild type level A** Pigmentation phenotype of the *D. shibae* wild type, Dshi_1135::Tn and complemented strain Dshi_1135::Tn, pRhoKS_Dshi_1135-Strep **B** Photopigments were extracted from bacteria grown in the dark and absorption was measured by UV/vis spectroscopy: *D. shibae* wild type (solid line), Dshi_1135::Tn (dashed line) and complemented strain Dshi_1135::Tn, pRhoKS_Dshi_1135-Strep (dotted line). Absorption at 370 and 780 nm is derived from bacteriochlorophyll, absorption at 500 nm from spheroidenone.

**Fig. S3**

**
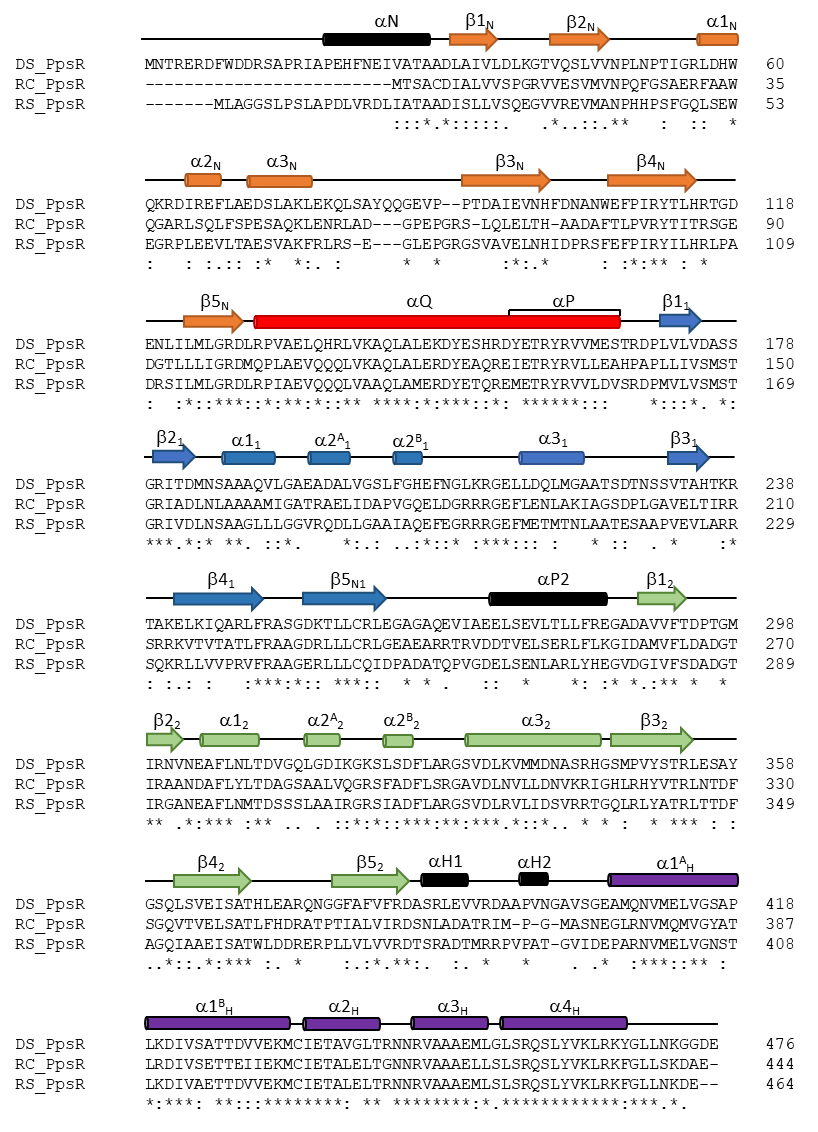
**

**Figure S2. Multiple sequence alignment of PpsR/ CrtJ proteins from *D. shibae* (Genbank Ac. ABV95264), *R. capsulatus* (Z11165) and *C. sphaeroides* (ABA79455). The alignment was performed using Clustal omega (Goujon et al., 2010; Sievers et al., 2011)** The secondary-structure elements are drawn according to protomer A of the PpsRΔHTH structure (PDB 4HH2) and a PSIPRED (McGuffin et al., 2000; Heintz et al., 2014) prediction of the secondary-structure elements in the HTH motif. Structural elements are colored as follows: N-domain PAS, orange; Q-linker , red; PAS1, blue; PAS2, green; HTH, purple.


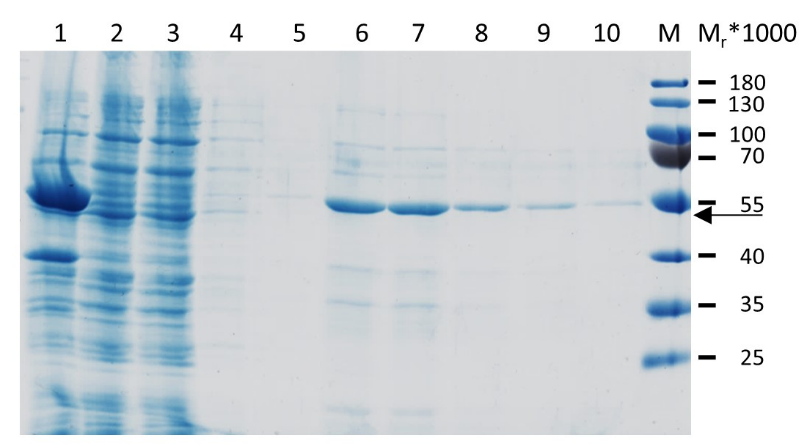
**Fig. S4**

**Figure S3. Affinity purification of recombinantly produced TRX-Strep-Dshi_1135 protein.** The TRX-StrepII-Dshi_1135 fusion protein was recombinantly overexpressed in *E. coli* BL21-CodonPlus(DE3)-RIL cells and subsequently purified *via* affinity chromatography using the Strep Tactin^®^ Superflow matrix. All samples were mixed with 2x SDS-loading dye, denatured at 95 °C for 10 min, subsequently analyzed on 12 % SDS gels and visualized *via* Coomassie Brilliant Blue staining. The arrow indicates the TRX-StrepII-Dshi_1135 protein with the calculated relative molecular mass of 51’650 Da. Lane 1, pellet fraction; lane 2, cell free extracts; lane 3, flow-through fraction, lane 4, washing step I; lane 5, washing step II. Lane 6, elution fraction I; lane 7, elution fraction II; lane 8, elution fraction III; lane 9, elution fraction IV; lane 10, elution fraction V; M, prestained molecular mass marker (relative molecular masses (*1’000) are indicated.

**
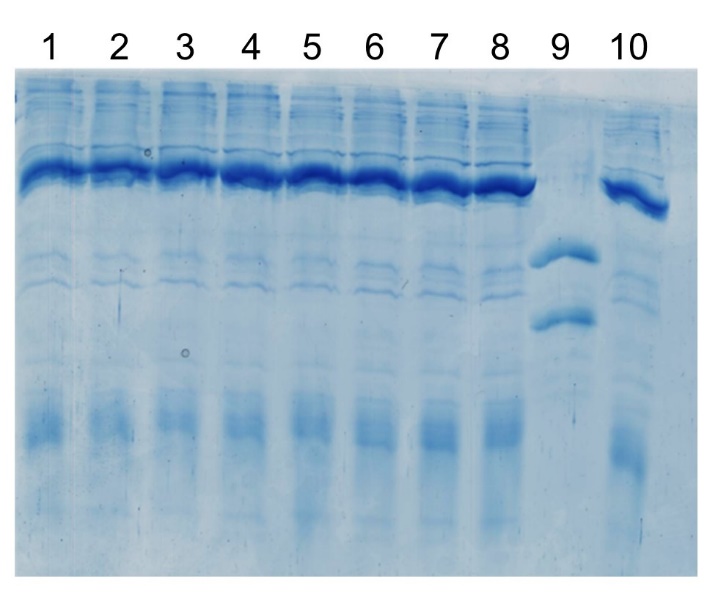
Fig. S4**

Figure S4: Phos-tag^TM^ SDS-gel after TRX-StrepII-Dshi_1135 autophosphorylation assay

Purified and reconstituted TRX-StrepII-Dshi_1135 protein was employed for an autophosphorylation assay, using Phos-tag^TM^ SDS-PAGE (FUJIFILM Wako Chemicals Europe, Neuss, Germany) (Barbieri and Stock, 2008). The protein was mixed with 1 µM, 10 µM, 100 µM or 1 mM ATP, respectively, and incubated overnight in a dark environment or under constant blue light (455 nm) irradiation. Reactions were quenched by addition of 2x SDS loading dye and samples were loaded onto 10 % Phos-tag^TM^ (100 µM) SDS gels. Protein bands were visualized by Coomassie Brilliant Blue staining. Lane 1: Dshi_1135 + 1 µM ATP, blue light. Lane 2: Dshi_1135 + 10 µM ATP, blue light. Lane 3: Dshi_1135 + 100 µM ATP, blue light. Lane 4: Dshi_1135 + 1 mM ATP, blue light. Lane 5: Dshi_1135 + 1 µM ATP, dark. Lane 6: Dshi_1135 + 10 µM ATP, dark. Lane 7: Dshi_1135 + 100 µM ATP, dark. Lane 8: Dshi_1135 + 1 mM ATP, dark. Lane 9: α-casein, positive control. Lane 10: Dshi_1135 untreated, negative control.

**Table S1:** Primers used for cloning of the expression plasmids for the bacterial two hybrid system using HiFi DNA assembly protocol (NEB, Ipswich, Massachusetts, USA)

| **Protein** | **Vektor** | **Primer Insert** | **Primer Vektor** |
| --- | --- | --- | --- |
| Dshi_1135 | pKT25_LOV | EH836  GGTCGACTCTAGAGGATatgaagccacgcacgcta  EH837  ACTTAGGTACCCGGGGtcaattgcccgcgggc | EH834  ATCCTCTAGAGTCGACC  EH835  CCCCGGGTACCTAAGT |
|  | pKNT25_LOV | EH836  GGTCGACTCTAGAGGATatgaagccacgcacgcta  EH839  GAGCTCGGTACCCGGGGattgcccgcgggcacg | EH834  ATCCTCTAGAGTCGACC  EH838  CCCCGGGTACCGAGCTC |
|  | pUT18_LOV | EH836  GGTCGACTCTAGAGGATatgaagccacgcacgcta  EH839  GAGCTCGGTACCCGGGGattgcccgcgggcacg | EH834  ATCCTCTAGAGTCGACC  EH838  CCCCGGGTACCGAGCTC |
|  | pUT18C_LOV | EH836  GGTCGACTCTAGAGGATatgaagccacgcacgcta  EH840  GAGCTCGGTACCCGGGGtcaattgcccgcgggc | EH834  ATCCTCTAGAGTCGACC  EH838  CCCCGGGTACCGAGCTC |
| PpsR | pKT25_PpsR | EH841  GGTCGACTCTAGAGGATatgaacacgcgggaaag  EH842  ACTTAGGTACCCGGGGctattcgtcgccgccct | EH834  ATCCTCTAGAGTCGACC  EH835  CCCCGGGTACCTAAGT |
|  | pKNT25_ PpsR | EH841  GGTCGACTCTAGAGGATatgaacacgcgggaaag  EH843  GAGCTCGGTACCCGGGGttcgtcgccgcccttg | EH834  ATCCTCTAGAGTCGACC  EH838  CCCCGGGTACCGAGCTC |
|  | pUT18_ PpsR | EH841  GGTCGACTCTAGAGGATatgaacacgcgggaaag  EH843  GAGCTCGGTACCCGGGGttcgtcgccgcccttg | EH834  ATCCTCTAGAGTCGACC  EH838  CCCCGGGTACCGAGCTC |
|  | pUT18C_ PpsR | EH841  GGTCGACTCTAGAGGATatgaacacgcgggaaag  EH844  GAGCTCGGTACCCGGGGctattcgtcgccgccct | EH834  ATCCTCTAGAGTCGACC  EH838  CCCCGGGTACCGAGCTC |
| PpaA | pKT25_PpaA | EH845  GGTCGACTCTAGAGGATatgtccaccccggaca  EH846  ACTTAGGTACCCGGGGttacgattcaagtcttgg | EH834  ATCCTCTAGAGTCGACC  EH835  CCCCGGGTACCTAAGT |
|  | pKNT25_PapA | EH845  GGTCGACTCTAGAGGATatgtccaccccggaca  EH847  GAGCTCGGTACCCGGGGcgattcaagtcttggttg | EH834  ATCCTCTAGAGTCGACC  EH838  CCCCGGGTACCGAGCTC |
|  | pUT18_PpaA | EH845  GGTCGACTCTAGAGGATatgtccaccccggaca  EH847  GAGCTCGGTACCCGGGGcgattcaagtcttggttg | EH834  ATCCTCTAGAGTCGACC  EH838  CCCCGGGTACCGAGCTC |
|  | pUT18C_PpaA | EH845  GGTCGACTCTAGAGGATatgtccaccccggaca  EH848  GAGCTCGGTACCCGGGGttacgattcaagtcttgg | EH834  ATCCTCTAGAGTCGACC  EH838  CCCCGGGTACCGAGCTC |

**Table S2.** Strains and plasmids used in this work

| **Strain** | **Description** | **Source/ /Reference** |
| --- | --- | --- |
| ***E. coli*** |  |  |
| DH10B | F^–^ *mcrA* Δ(*mrr*-*hsd*RMS-*mcr*BC) Φ80*lac*ZΔM15 Δ*lac*X74 *rec*A1 *end*A1 *ara*D139 Δ(*ara leu*) 7697 *gal*U *gal*K *rps*L *nup*G λ^–^ | Invitrogen (Carlsbad, CA, USA) |
| BL21(DE3) | *fhuA2 [lon] ompT gal (λ DE3) [dcm] ∆hsdS λ DE3 = λ sBamHIo ∆EcoRI-B int::(lacI::PlacUV5::T7 gene1) i21 ∆nin5* | New England Biolabs (Ipswich, MA, USA) |
| BL21-CodonPlus (DE3)-RIL | *E. coli B F^–^ ompT hsdS(r_B_ ^–^ m_B_ ^–^ ) dcm^+^ Tet^r^ gal λ(DE3) endA Hte [argU ileY leuW Cam^r^ ]* | Stratagene (Santa Clara, CA (USA) |
| Novablue (DE3) | *endA1 hsdR17(rK12-mK12+) supE44 thi-1 recA1 gyrA96 relA1 lac F’[proA+B+ laclqZΔM15::Tn10] (TetR)* | Merck (Darmstadt, Deutschland) |
| ST18 | *E. coli* S17-1*ΔhemA thi pro hsdR*-M- chromosomal integrated [*RP4-2 Tc::Mu:Km^r^::Tn7, Tra+ Trir Strr*] | (Thoma and Schobert, 2009) |
| BTH101 | *F-, cya-99, araD139, galE15, galK16, rpsL1, (Str^r^), hsdR2, mcrA1, mcrB1* | Euromedex (Souffelweyers-heim, France) |
| BACTH 31 | BTH101; pUT18-*ppsR* (PpsR-T18), Amp^r;^ pKNT25, Kan^r^ | This work |
| BACTH 32 | BTH101; pUT18C-*ppsR*(T18-PpsR), Amp^r^; pKNT25, Kan^r^ | This work |
| BACTH 33 | BTH101; pUT18-Dshi_1135 (Dshi_1135-T18), Amp^r^; pKNT25, Kan^r^ | This work |
| BACTH 34 | BTH101; pUT18C-Dshi_1135(T18-Dshi_1135), Amp^r^; pKNT25, Kan^r^ | This work |
| BACTH 35 | BTH101; pUT18, Amp^r;^ pKNT25, Kan^r^ | This work |
| BACTH 36 | BTH101; pUT18C, Amp^r;^ pKNT25, Kan^r^ | This work |
| BACTH 43 | BTH101; pUT18-*ppsR* (PpsR-T18), Amp^r^; pKT25, Kan^r^ | This work |
| BACTH 44 | BTH101; pUT18C-*ppsR* (T18-PpsR), Amp^r^; pKT25, Kan^r^ | This work |
| BACTH 45 | BTH101; pUT18-Dshi_1135 (Dshi_1135-T18); Amp^r^; pKT25, Kan^r^ | This work |
| BACTH 46 | BTH101; pUT18C-Dshi_1135 (T18-Dshi_1135); Amp^r^; pKT25, Kan^r^ | This work |
| BACTH 47 | BTH101; pUT18, Amp^r^; pKT25, Kan^r^ | This work |
| BACTH 48 | BTH101; pUT18C, Amp^r;^ pKT25, Kan^r^ | This work |
| BACTH 67 | BTH101; pUT18, Amp^r^; pKNT25-*ppsR* (PpsR-T25), Kan^r^ | This work |
| BACTH 68 | BTH101; pUT18C, Amp^r^; pKNT25-*ppsR* (PpsR-T25), Kan^r^ | This work |
| BACTH 71 | BTH101; pUT18, Amp^r^; pKT25-*ppsR* (T25-PpsR), Kan^r^ | This work |
| BACTH 72 | BTH101; pUT18C, Amp^r^; pKT25-*ppsR* (T25-PpsR), Kan^r^ | This work |
| BACTH 75 | BTH101; pUT18, Amp^r^; pKNT25-Dshi_1135 (Dshi_1135-T25), Kan^r^ | This work |
| BACTH 76 | BTH101; pUT18C, Amp^r^; pKNT25-Dshi_1135 (Dshi_1135-T25), Kan^r^ | This work |
| BACTH 79 | BTH101; pUT18, Amp^r^; pKT25-Dshi_1135 (T25-Dshi_1135), Kan^r^ | This work |
| BACTH 80 | BTH101; pUT18C, Amp^r^; pKT25-Dshi_1135 (T25-Dshi_1135), Kan^r^ | This work |
| BACTH 81 | BTH101; pKT25-*ppsR* (T25-PpsR) Kan^r^; pUT18-*ppsR* (PpsR-T18) Amp^r^; | This work |
| BACTH 82 | BTH101; pKNT25-*ppsR* (PpsR-T25) Kan^r^; pUT18-*ppsR* (PpsR-T18) Amp^r^; | This work |
| BACTH 83 | BTH101; pKT25-*ppsR* (T25-PpsR) Kan^r^; pUT18C-*ppsR*(T18-PpsR) Amp^r^; | This work |
| BACTH 84 | BTH101; pKNT25-*ppsR* (PpsR-T25) Kan^r^; pUT18C-*ppsR*(T18-PpsR), Amp^r^; | This work |
| BACTH 85 | BTH101; pUT18-Dshi_1135 (Dshi_1135-T18), Amp^r^; pKT25-Dshi_1135 (T25-Dshi_1135), Kan^r^ | This work |
| BACTH 86 | BTH101; pUT18-Dshi_1135 (Dshi_1135-T18), Amp^r^; pKNT25-Dshi_1135 (Dshi_1135-T25), Kan^r^ | This work |
| BACTH 87 | BTH101; pUT18C-Dshi_1135 (T18-Dshi_1135), Amp^r^; pKT25-Dshi_1135 (T25-Dshi_1135), Kan^r^ | This work |
| **Strain** | **Description** | **Source/ /Reference** |
| BACTH 88 | BTH101; pUT18C-Dshi_1135 (T18-Dshi_1135), Amp^r^; pKNT25-Dshi_1135 (Dshi_1135-T25), Kan^r^ | This work |
| BACTH 89 | BTH101; pUT18-Dshi_1135 (Dshi_1135-T18), Amp^r^; pKT25-*ppsR* (T25-PpsR) Kan^r^ | This work |
| BACTH 90 | BTH101; pUT18-Dshi_1135 (Dshi_1135-T18), Amp^r^; pKNT25-*ppsR* (PpsR-T25) Kan^r^ | This work |
| BACTH 91 | BTH101; pUT18C-Dshi_1135(T18-Dshi_1135), Amp^r^; pKT25-*ppsR* (T25-PpsR) Kan^r^ | This work |
| BACTH 92 | BTH101; pKT25-Dshi_1135 (T25-Dshi_1135), Kan^r^; pUT18-*ppsR* (PpsR-T18), Amp^r^; | This work |
| BACTH 93 | BTH101; pKNT25-Dshi_1135 (Dshi_1135-T25), Kan^r^; pUT18-*ppsR* (PpsR-T18), Amp^r^ | This work |
| BACTH 94 | BTH101; pKT25-Dshi_1135 (T25-Dshi_1135), Kan^r^; pUT18C-*ppsR*(T18-PpsR), Amp^r^ | This work |
| BACTH 95 | BTH101; pKNT25-Dshi_1135 (Dshi_1135-T25), Kan^r^; pUT18C-*ppsR*(T18-PpsR), Amp^r^ | This work |
| BACTH 96 | BTH101; pKT25-*ppaA* (T25-PpaA), Kan^r^; pUT18-*ppaA* (PpaA-T18), Amp^r^; | This work |
| BACTH 97 | BTH101; pKNT25-*ppaA* (PpaA-T25), Kan^r^; pUT18-*ppaA* (PpaA-T18), Amp^r^; | This work |
| BACTH 98 | BTH101; pKT25-*ppaA* (T25-PpaA), Kan^r^; pUT18C-*ppaA* (T18-PpaA), Amp^r^; | This work |
| BACTH 99 | BTH101; pKNT25-*ppaA* (PpaA-T25), Kan^r^; pUT18C-*ppaA* (T18-PpaA), Amp^r^; | This work |
| BACTH 100 | BTH101; pUT18-*ppaA* (PpaA-T18), Amp^r^; pKT25, Kan^r^ | This work |
| BACTH 101 | BTH101; pUT18-*ppaA* (PpaA-T18), Amp^r^; pKNT25, Kan^r^ | This work |
| BACTH 102 | BTH101; pUT18C-*ppaA* (T18-PpaA), Amp^r^; pKT25, Kan^r^ | This work |
| BACTH 103 | BTH101; pUT18C-*ppaA* (T18-PpaA), Amp^r^; pKNT25, Kan^r^ | This work |
| BACTH 104 | BTH101; pUT18, Amp^r^; pKT25-*ppaA* (T25-PpaA), Kan^r^ | This work |
| BACTH 105 | BTH101; pUT18, Amp^r^; pKNT25-*ppaA* (PpaA-T25), Kan^r^ | This work |
| BACTH 106 | BTH101; pUT18C, Amp^r^; pKT25-*ppaA* (T25-PpaA), Kan^r^ | This work |
| BACTH 107 | BTH101; pUT18C, Amp^r^; pKNT25-*ppaA* (PpaA-T25), Kan^r^ | This work |
| BACTH 108 | BTH101; pKT25-*ppsR* (T25-PpsR) Kan^r^; pUT18-*ppaA* (PpaA-T18), Amp^r^ | This work |
| BACTH 109 | BTH101; pKNT25-*ppsR* (PpsR-T25) Kan^r^; pUT18-*ppaA* (PpaA-T18), Amp^r^ | This work |
| BACTH 110 | BTH101; pKT25-*ppsR* (T25-PpsR) Kan^r^; pUT18C-*ppaA* (T18-PpaA), Amp^r^ | This work |
| BACTH 111 | BTH101; pKNT25-*ppsR* (PpsR-T25) Kan^r^; pUT18C-*ppaA* (T18-PpaA), Amp^r^ | This work |
| BACTH 112 | BTH101; pKT25-*ppaA* (T25-PpaA), Kan^r^; pUT18-*ppsR* (PpsR-T18), Amp^r^ | This work |
| BACTH 113 | BTH101; pKNT25-*ppaA* (PpaA-T25), Kan^r^; pUT18-*ppsR* (PpsR-T18), Amp^r^ | This work |
| BACTH 114 | BTH101; pKT25-*ppaA* (T25-PpaA), Kan^r^; pUT18C-*ppsR*(T18-PpsR), Amp^r^ | This work |
| BACTH 115 | BTH101; pKNT25-*ppaA* (PpaA-T25), Kan^r^, pUT18C-*ppsR*(T18-PpsR), Amp^r^ | This work |
| BACTH 116 | BTH101; pKT25-Dshi_1135 (T25-Dshi_1135), Kan^r^; pUT18-*ppaA* (PpaA-T18), Amp^r^ | This work |
| BACTH 117 | BTH101; pKNT25-Dshi_1135 (Dshi_1135-T25), Kan^r^, pUT18-*ppaA* (PpaA-T18), Amp^r^ | This work |
| BACTH 118 | BTH101; pKT25-Dshi_1135 (T25-Dshi_1135), Kan^r^; pUT18C-*ppaA* (T18-PpaA), Amp^r^ | This work |
| BACTH 119 | BTH101; pKNT25-Dshi_1135 (Dshi_1135-T25), Kan^r^; pUT18C-*ppaA* (T18-PpaA), Amp^r^ | This work |
| BACTH 120 | BTH101; pUT18-Dshi_1135 (Dshi_1135-T18), Amp^r^; pKT25-*ppaA* (T25-PpaA), Kan^r^ | This work |
| BACTH 121 | BTH101; pUT18-Dshi_1135 (Dshi_1135-T18), Amp^r^; pKNT25-*ppaA* (PpaA-T25), Kan^r^ | This work |
| BACTH 122 | BTH101; pUT18C-Dshi_1135(T18-Dshi_1135), Amp^r^; pKT25-*ppaA* (T25-PpaA), Kan^r^ | This work |
| BACTH 123 | BTH101; pUT18C-Dshi_1135(T18-Dshi_1135), Amp^r^; pKNT25-*ppaA* (PpaA-T25), Kan^r^ | This work |
| BACTH 124 | BTH101; pUT18C-*zip*, T18-Zip, Amp^r^; pKT25-*zip*, T25-Zip, Kan^r^ | This work |
| **Strain** | **Description** | **Source/ /Reference** |
| ***D. shibae*** |  |  |
| DFL12^T^ | wild type, isolated from the dinoflagellate *Prorocentrum lima*, type strain, DSM16493^T^ | (Biebl et al., 2005) |
| DSTn2323 | Dshi_ 3544(*acsF*)::Tn*,* Gm^r^ | (Ebert et al., 2013) |
| DSTn12510 | Dshi_1135::Tn, Gm^r^ | (Ebert et al., 2013) |
| DSTn11705 | Dshi_1387(*clpX)*::Tn, Gm^r^ | (Ebert et al., 2013) |
| DSTn4031 | Dshi_3532(*ppaA*)::Tn, Gm^r^ | (Ebert et al., 2013) |
| DSTn4634 | Dshi_3531(*ppsR*)::Tn, Gm^r^ | (Ebert et al., 2013) |
|  |  |  |
| DS181 | DFL12^T^; pBBR1LIC_*bchF*-*lacZ*, Cm^r^ | This work |
| DS187 | *ppaA*::Tn, (Dshi_3532), Gm^r^; pBBR1LIC_*bchF*-*lacZ*, Cm^r^ | This work |
| DS188 | Dshi_1135::Tn, Gm^r^; pBBR1LIC_*bchF*-*lacZ*, Cm^r^ | This work |
| DS189 | *ppsR*::Tn, (Dshi_3531), Gm^r^; pBBR1LIC_*bchF*-*lacZ*, Cm^r^ | This work |
| DS190 | DFL12^T^; pBBR1LIC_*bchF(*mut)-*lacZ*, mutations in potential PpsR binding sites at positions -79/-77 from TGT to CCA and -30/-28 from ACA to TGG, Cm^r^ | This work |
| DS266 | Dshi_1135::Tn, Gm^r^; pRhokS_Dshi_1135-Strep*, Cm*^r^ | This work |
|  |  |  |
| **Plasmids** |  |  |
|  | pET52b(+)_Trx/StrepII | (Frädrich et al., 2012) |
|  | pET52b(+)_Trx/StrepII-Dshi_1135 | This work |
|  | pBBR1LIC_*bchF*-*lacZ, cm^r^* | This work |
|  | pBBR1LIC_*bchF(mut)*-*lacZ, cm^r^* | This work |
|  | pRhoKS | (Katzke et al., 2010) |
|  | pRhoKS_Dshi_1135-StrepII | This work |
|  | pKT25 | (Karimova et al., 2001) |
|  | pKNT25 | (Karimova et al., 2001) |
|  | pUT18 | (Karimova et al., 2001) |
|  | pUT18C | (Karimova et al., 2001) |
|  |  |  |

**References**

Biebl, H., Allgaier, M., Tindall, B.J., Koblizek, M., Lunsdorf, H., Pukall, R., et al. (2005). Dinoroseobacter shibae gen. nov., sp. nov., a new aerobic phototrophic bacterium isolated from dinoflagellates. *International journal of systematic and evolutionary microbiology* 55(Pt 3)**,** 1089-1096. doi: 10.1099/ijs.0.63511-0.

Ebert, M., Laass, S., Burghartz, M., Petersen, J., Kossmehl, S., Wohlbrand, L., et al. (2013). Transposon mutagenesis identified chromosomal and plasmid genes essential for adaptation of the marine bacterium Dinoroseobacter shibae to anaerobic conditions. *Journal of bacteriology* 195(20)**,** 4769-4777. doi: 10.1128/JB.00860-13.

Frädrich, C., March, A., Fiege, K., Hartmann, A., Jahn, D., and Härtig, E. (2012). The transcription factor AlsR binds and regulates the promoter of the alsSD operon responsible for acetoin formation in Bacillus subtilis. *J Bacteriol* 194(5)**,** 1100-1112. doi: 10.1128/jb.06425-11.

Goujon, M., McWilliam, H., Li, W., Valentin, F., Squizzato, S., Paern, J., et al. (2010). A new bioinformatics analysis tools framework at EMBL–EBI. *Nucleic Acids Research* 38(suppl_2)**,** W695-W699. doi: 10.1093/nar/gkq313.

Heintz, U., Meinhart, A., and Winkler, A. (2014). Multi-PAS domain-mediated protein oligomerization of PpsR from Rhodobacter sphaeroides. *Acta Crystallogr D Biol Crystallogr* 70(Pt 3)**,** 863-876. doi: 10.1107/s1399004713033634.

Karimova, G., Ullmann, A., and Ladant, D. (2001). Protein-protein interaction between Bacillus stearothermophilus tyrosyl-tRNA synthetase subdomains revealed by a bacterial two-hybrid system. *J Mol Microbiol Biotechnol* 3(1)**,** 73-82.

Katzke, N., Arvani, S., Bergmann, R., Circolone, F., Markert, A., Svensson, V., et al. (2010). A novel T7 RNA polymerase dependent expression system for high-level protein production in the phototrophic bacterium Rhodobacter capsulatus. *Protein Expression and Purification* 69(2)**,** 137-146. doi: http://dx.doi.org/10.1016/j.pep.2009.08.008.

McGuffin, L.J., Bryson, K., and Jones, D.T. (2000). The PSIPRED protein structure prediction server. *Bioinformatics* 16(4)**,** 404-405. doi: 10.1093/bioinformatics/16.4.404.

Sievers, F., Wilm, A., Dineen, D., Gibson, T.J., Karplus, K., Li, W., et al. 2011. Fast, scalable generation of high-quality protein multiple sequence alignments using Clustal Omega. *Molecular systems biology* [Online], 7. Available: http://europepmc.org/abstract/MED/21988835

Thoma, S., and Schobert, M. (2009). An improved Escherichia coli donor strain for diparental mating. *FEMS microbiology letters* 294(2)**,** 127-132.
